# Role of *ZFHX4* in orofacial clefting based on human genetic data and zebrafish models

**DOI:** 10.1038/s41431-024-01775-9

**Published:** 2024-12-19

**Authors:** Nina Ishorst, Selina Hölzel, Carola Greve, Öznur Yilmaz, Tobias Lindenberg, Jessica Lambertz, Dmitriy Drichel, Berina Zametica, Enrico Mingardo, Jeshurun C. Kalanithy, Khadija Channab, Duygu Kibris, Sabrina Henne, Franziska Degenhardt, Anna Siewert, Michael Dixon, Teresa Kruse, Edwin Ongkosuwito, Katta M. Girisha, Shruti Pande, Stefanie Nowak, Gregor Hagelueken, Matthias Geyer, Carine Carels, Iris A.L.M. van Rooij, Kerstin U. Ludwig, Benjamin Odermatt, Elisabeth Mangold

**Affiliations:** 1 Institute of Human Genetics, University of Bonn, School of Medicine & University Hospital Bonn, Bonn, Germany; 2 Institute of Anatomy, Division of Neuroanatomy, University of Bonn, School of Medicine & University Hospital Bonn, Bonn, Germany; 3 Institute of Anatomy and Cell Biology, University of Bonn, School of Medicine & University Hospital Bonn, Bonn, Germany; 4 Cologne Center for Genomics, University of Cologne, Cologne, Germany; 5 Faculty of Biology, Medicine & Health, University of Manchester, Manchester M13 9PL, UK; 6 University of Cologne, Faculty of Medicine and University Hospital Cologne, Department of Orthodontics, Cologne, Germany; 7 Department of Dentistry, Section of Orthodontics and Craniofacial Biology, Radboud Institute for Health Sciences, Radboud University Medical Center, Nijmegen, The Netherlands; 8 Department of Medical Genetics, Kasturba Medical College, Manipal, Manipal Academy of Higher Education, Manipal, India; 9 Institute of Structural Biology, University of Bonn, Bonn, Germany; 10 Department of Human Genetics, KU Leuven, Leuven, Belgium; 11 IQ Health Science Department, Radboud University Medical Center, Nijmegen, The Netherlands

## Abstract

Orofacial clefting (OFC) is a frequent congenital anomaly and can occur either in the context of underlying syndromes or in isolation (nonsyndromic). The two common OFC phenotypes are cleft lip with/without cleft palate (CL/P) and cleft palate only (CPO). In this study, we searched for penetrant CL/P genes, by evaluating de novo copy number variants (CNV) from an exome sequencing dataset of 50 nonsyndromic patient-parent trios. We detected a heterozygous 86 kb de novo deletion affecting exons 4–11 of *ZFHX4*, a gene previously associated with OFC. Genetic and phenotypic data from our in-house and the AGORA cohort (710 and 229 individuals with nonsyndromic CL/P) together with literature and database reviews demonstrate that *ZFHX4* variants can lead to both nonsyndromic and syndromic forms not only of CL/P but also CPO. Expression analysis in published single-cell RNA-sequencing data (mouse embryo, zebrafish larva) at relevant time-points support an important role of *Zfhx4/zfhx4* in craniofacial development. To characterize the role of *zfhx4* in zebrafish craniofacial development, we knocked out/down the zebrafish orthologue. Cartilage staining of the *zfhx4* CRISPR F0 knockout and morpholino knockdown at 4 days post-fertilization showed an underdeveloped and abnormally shaped ethmoid plate and cartilaginous jaw (resembling micrognathia). While there is evidence for the dominant inheritance of *ZFHX4* variants in OFC, we here present a patient with a possible recessive inheritance. In conclusion, *ZFHX4* has a highly heterogeneous phenotypic spectrum and variable mode of inheritance. Our data highlight that *ZFHX4* should be considered in genetic testing in patients with nonsyndromic clefting.

## Introduction

Orofacial clefts are among the most common congenital malformations in humans [1]. The two common phenotypes are cleft lip with or without cleft palate (CL/P) and cleft palate only (CPO). Clefting can occur in the context of complex syndromes or in an isolated nonsyndromic form, which is more common, i.e. it is assumed that 70% of CL/Ps are nonsyndromic (nsCL/P [MIM:612858]) [2]. The boundaries between mild syndromic and nonsyndromic forms can be fluid, especially when additional anomalies are continuous features, such as intellectual disability. Genetic and epidemiological data on nsCL/P indicate that high-penetrance genetic variants in “major” genes act on a multi-factorial background [3]. Genome-wide association studies (GWAS) and follow-up studies have identified more than 40 common risk loci across diverse populations [4–7]. However, the estimated heritability is around 90% and GWAS-based estimates indicate that common variation explains only a fraction of the estimated heritability (e.g., <40% among Europeans) [6].

A fraction of the missing heritability of nsCL/P may be explained by rare, highly penetrant variants in genes potentially involved in craniofacial development. Sequencing studies have found functionally relevant penetrant variants in genes that were selected as candidate genes on the basis of evidence from syndromic forms of orofacial clefting [8, 9] or their location at previously identified linkage/GWAS loci [10–12]. More systematic approaches identified novel, highly penetrant nsCL/P susceptibility genes via exome sequencing (ES) in multiple affected families [13].

A complementary approach to the identification of highly penetrant susceptibility genes is to search for rare, highly penetrant de novo variants [14–16]. A study of nsCL/P and nonsyndromic cleft palate only (nsCPO) identified a significant enrichment of loss-of-function de novo variants in two genes, one of which was Zinc Finger Homeobox 4 (*ZFHX4)* [14]. A functional study in mice provided additional evidence for *ZFHX4* as an OFC gene [17].

Recent studies have focused mainly on the detection of variants that affect single bases or small indels in candidate genes. In this study, we identified highly penetrant candidate genes for nsCL/P by detecting large de novo copy number variants (CNV) through reanalysis of ES data of 50 patient-parent trios. This revealed a de novo heterozygous 86 kb deletion in *ZFHX4*. We used a targeted-sequencing approach to evaluate the role of *ZFHX4* in an independent cohort of individuals with nsCL/P. Further, we characterized the effects of *zfhx4* disruption on embryonic development of the craniofacial region in zebrafish larvae (zfl). Finally, to evaluate the clinical relevance of our findings, we compiled data from an unpublished syndromic patient and from literature searches and publicly available databases.

## Materials and Methods

### Patient-parent trio dataset

The patient-parent trio dataset included ES data of 50 patients with nsCL/P and their unaffected parents of Central European ancestry, as described by Ishorst et al. [15].

### CNV calling and filtering

**Fig. 1:**
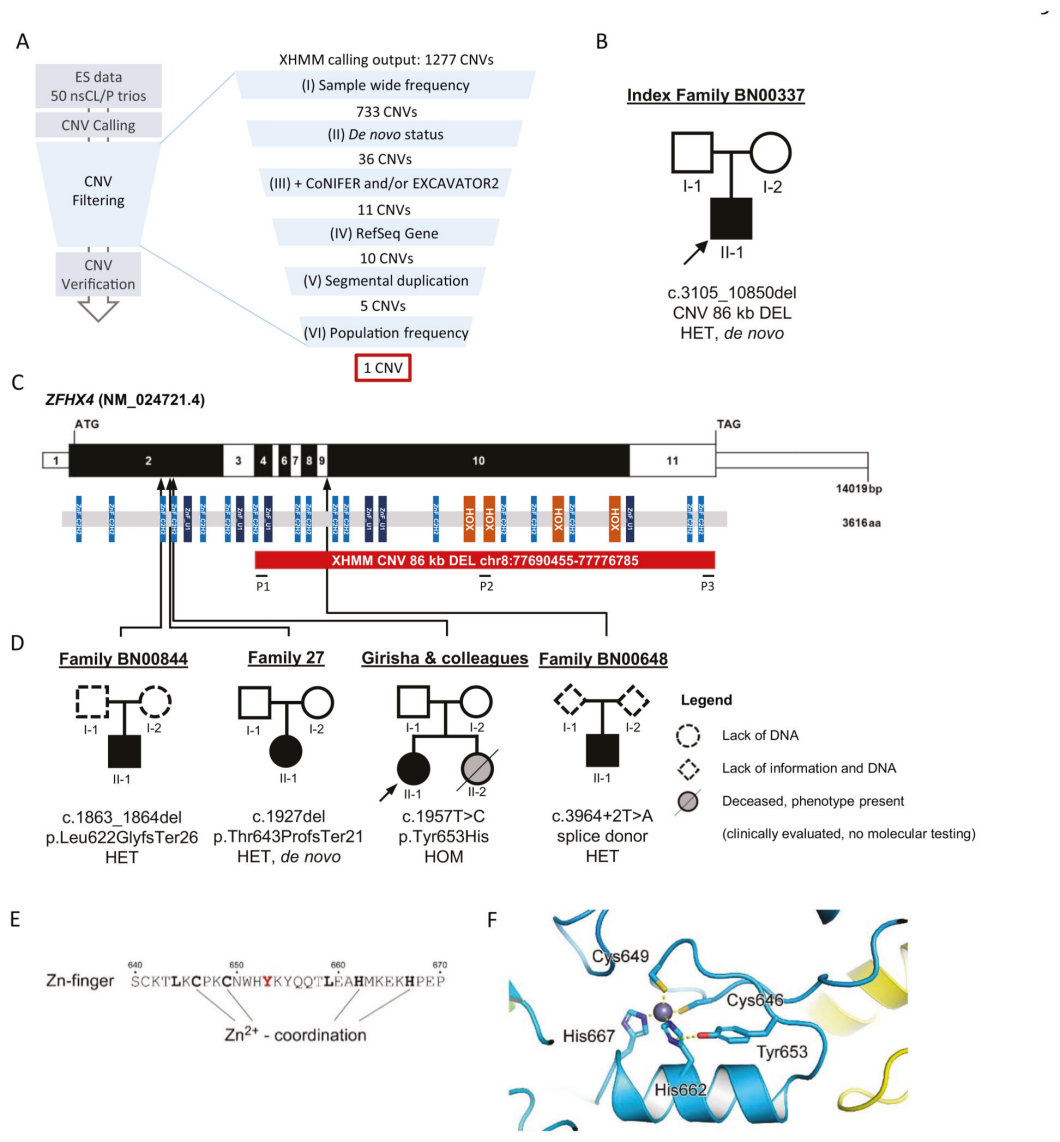
Workflow of the CNV study and additional individuals/families with *ZFHX4* variants. **A** Workflow of the CNV study. CNVs were called from an existing dataset including exome sequencing data for 50 nsCL/P trios (Ishorst et al. 2022). CNV filtering was performed as follows. (I) The CNV frequency threshold was set to ≤10% among all 50 nsCL/P trios. (II) De novo CNV calling was performed using PLINK/Seq. (III) Only CNVs that were extracted using either CoNIFER and/or EXCAVATOR2 in addition to XHMM were retained in the analysis. CNVs that (IV) did not span RefSeq Genes, (V) overlapped by >50% with regions of segmental duplication or >80% with genes in difficult to analyze regions (Segmental Dups track, UCSC Genome Browser on Human Feb. 2009 (GRCh37/hg19) Assembly), and (VI) had population frequencies ≥0.01 were excluded. **B** Pedigree of index trio with heterozygous de novo CNV in *ZFHX4*. **C** Exon and protein domain structure of human *ZFHX4*. Exons are colored in alternating white and black, and positions of the start codon (ATG) and stop codon (TAG) are marked. ZFHX4 is a transcription factor with four homeodomains (orange) and 23 zinc finger domains (blue). Protein domains are positioned in proportion to the corresponding exons. Red bar, heterozygous *ZFHX4* de novo deletion in the index patient with nsCLP. P1-P3, position of primer pairs spanning the CNV for qPCR verification; black arrows, positions of additional *ZFHX4* variants including loss-of-function variants from targeted-sequencing and a homozygous missense variant from a collaboration with Girisha & colleagues. **D** Pedigrees of four additional families (targeted-sequencing study and collaboration with Girisha & colleagues). **E** Tyr653 is one out of seven conserved residues (drawn bold) in the fold of a canonical zinc-finger motif. **F** Model of the zinc-finger structure in ZFHX4 proposes the formation of a hydrogen bond between Tyr653 and His662. Mutation of Tyr653 to histidine could change this conformational arrangement of side chains and thus impair zinc-binding. CNV copy number variation, nsCL/P nonsyndromic cleft lip with/without cleft palate, qPCR quantitative polymerase chain reaction, ES exome sequencing.

CNV calling from ES BAM files (Fig. 1A) was performed using three read-count based CNV detection tools: XHMM (eXome hidden Markov model) [18], CoNIFER (copy number inference from exome reads) [19], and EXCAVATOR2 [20]. For the latter, parental samples were pooled and utilized for read-count normalization and CNVs were called from patients only.

XHMM was used as the primary algorithm and, with minor changes, filtering recommendations given in basic protocol 3 of the XHMM manuscript were followed [21] (Fig. 1A, I-III). Common XHMM calls with frequencies of >10% among all nsCL/P trios were excluded. CNVs that overlapped by more than 80% were considered the same CNV. Next, de novo CNV calling on XHMM calls was performed using PLINK/Seq (quality score threshold SQ = 60). Filtering was continued for CNVs that were also detected by at least one of the secondary algorithms (Fig. 1A IV-VI). The AnnotSV web browser [22] was used for CNV population frequency annotation based on Genome Aggregation Database (gnomAD), DGV, and 1000 Genomes. For all variants, plausibility was visually inspected using the Integrative Genomics Viewer (IGV) v.2.3.98 [23].

### CNV validation with quantitative PCR

The identified CNV was experimentally validated using quantitative polymerase chain reaction (qPCR). Utilizing Primer3 version 4.1.0, three primer pairs were designed with product sizes of 120 to 150 bp covering the 5′- and 3′-end and the center of the CNV. Two control primer pairs spanning the human housekeeping genes basonuclin 1 (*BNC1*) and cystic fibrosis transmembrane conductance factor (*CFTR*) were included (for primer sequences, refer to Table S1). Light Cycler® SYBR Green I Master was used for relative quantification in triplicates on a Light Cycler® 480 (both: Roche, Basel, Switzerland). Cycle threshold values (C_T_) had to be < 35 to be included. If a triplicate C_T_ standard deviation exceeded 0.2, the most divergent value was excluded from analyses. For copy number determination, we applied the ΔΔC_T_ method [24]. *BNC1* and *CFTR* were used for normalization. The DNAs of two randomly allocated unaffected parents from the patient-parent ES cohort not carrying the CNV of interest according to the three CNV calling algorithms were used as references.

### Targeted-sequencing

The targeted-sequencing cohort included 710 individuals with nsCL/P from a Central European cohort from Bonn (Germany), 229 individuals with nsCL/P from the AGORA cohort (Netherlands), and 845 population-matched control individuals [25, 26]. All individuals were phenotypically well characterized. *ZFHX4* was part of a larger targeted-sequencing panel of 14 genes evaluated using single-molecule molecular inversion probes (smMIPs) (unpublished). In short, the smMIP assay design was performed using an in-house version of MIPgen, as described previously [15]. Library preparation followed standard procedures with minor modifications [15]. Final libraries were sequenced on the Illumina NovaSeq6000 platform (S2, 2 × 150 bp). Base call files were demultiplexed and converted into FASTQ files using bcl2fastq. Reads were aligned to GRCh37/hg19 using BWA-MEM. Trimming and collapsing of reads were performed using MIPgen scripts as described elsewhere [15]. Collapsing reads that originate from the same starting molecule (identified by a molecular tag) into a single strand consensus sequence allows correction for errors introduced by PCR amplification. The coverages per sample and per exon were calculated using samtools 0.1.19. Samples with an average collapsed coverage of <50× and exons with an average coverage of <20× were excluded. Variant calling was performed using UnifiedGenotyper and annotations were done using Ensembl Variant Predictor (VEP). The gnomAD was used to assign the population frequencies of variants. When DNA was available, the de novo status was determined by Sanger sequencing.

### ZFHX4 protein structure modeling

The protein structure of human ZFHX4 (UniProt accession code Q86UP3; 3567 aa) was modeled with AlphaFold3, adding 20 zinc atoms as optional ligands [27]. Protein graphics were generated using the PyMOL Molecular Graphics System (Version 2.5.5 Schrödinger, LLC).

### Rare variant association testing for *ZFHX4*

Rare variant association for *ZFHX4* was performed using SKAT-O (SKAT package v2.0.0) suitable for gene-based multiple variant tests. For the analysis a genotype matrix, and vectors of binary phenotypes were prepared.

### Assessment of ZFHX4 conservation and *Zfhx4/zfhx4* expression

Conservation of ZFHX4 at the amino acid level between human, mouse, and zebrafish (zf) was assessed using Clustal Omega from EMBL-EBI [28]. *Zfhx4* expression was inspected in two mouse RNA-Seq datasets representing the period of craniofacial development. A bulk RNA-Seq dataset from murine secondary palatal shelves (E10.5 to E14.5) (*BioStudies* accession number E-MTAB-3157) and single-cell RNA-Seq (scRNA-Seq) datasets from whole mouse embryos from E9.5 to E13.5 and mouse embryonic facial tissues at E11.5 were analyzed [29]. Further, *zfhx4* expression was evaluated using a publicly available in situ hybridization dataset and a scRNA-Seq Atlas of zfl development (Zebrahub) at 30 to 48 h post-fertilization and 1 to 3 days post-fertilization (dpf), representing the zfl period of craniofacial development [30, 31]. Finally, we compared the expression of *Zfhx4/zfhx4* with mouse/zebrafish (zf) orthologues of three established OFC genes, *CDH1, IRF6*, and *GRHL3* [13, 32, 33].

### Zebrafish husbandry and embryo maintenance

*Danio rerio* (zebrafish, zf) were kept according to national laws and recommendations by Westerfield [34]. Zfl of the wild-type AB/TL strain acquired by natural fish spawning in the morning and raised at 28 °C in Danieau (30%) medium on a 14 h light to 10 h dark cycle were used for all experiments. To inhibit pigmentation, zfl were treated with PTU (1-phenyl-2-thiourea, final concentration 0.003% in Danieau) from 1 dpf. All experimental groups were kept at the same density, temperature/light conditions and medium change cycles. All fish experiments were carried out before 5 dpf.

### Microinjections for morpholino oligonucleotide *zfhx4* knockdown (MO-KD)

A morpholino oligonucleotide (GeneTools, LLC, Philomath, OR, USA) targeting the *zfhx4* translational start site was used for the specific knockdown of all three transcripts (MO-KD). Zebrafish eggs were collected within 20 min after breeding and the yolk of one-cell staged embryos was pressure injected with 6 ng of MO-KD (1.7 nl/embryo) and 6 ng of standard control MO (Ctrl-MO). Uninjected embryos (UI_MO_) were kept as controls.

### Microinjections for *zfhx4* CRISPR/Cas9 F0-knockout (F0-KO)

All substances were ordered from Integrated DNA Technologies (IDT, Leuven, Belgium). Five crRNAs targeting exon 3 of *zfhx4* were designed using CRISPRscan [35] and equal amounts of each crRNA were combined to obtain a 100 μM crRNA stock. Then, 3 μl of crRNA stock and 3 μl of tracrRNA (100 μM) were diluted in 44 μl of nuclease-free IDT duplex buffer and annealed to form gRNAs at 95 °C for 5 min. gRNAs (6 μl) were incubated with equal volumes of Cas9 protein (1 μg/μl, diluted in PBS) at 37 °C for 10 min. Once cooled to room temperature, 1 μl of Phenol red was added. As a negative control, a mixture of three crRNAs not matching any genomic locus (Scrambled control, Ctrl-Scr) was prepared as described above [36]. Then, ∼1.7 nl Cas9/gRNA/phenol red mix was pressure injected per one-cell staged zebrafish embryo (see Table S1 for gRNA sequences).

### DNA extraction and PCR

To determine the efficiency of F0-KO, DNA was isolated from 10–15 zfl at 4 dpf from (i) zfl injected with the F0-KO mix, (ii) zfl injected with the Ctrl-Scr mix, and (iii) uninjected zfl (UI_CRISPR_). DNA was isolated following Meeker et al. [37] using 30 μl of 50 mM NaOH per zfl. PCR was performed using 2 μl of DNA. PCR primers were designed to bind to introns adjacent to exon 3 and to amplify a product of 720 bp (see Table S1 for primer sequences).

### Protein isolation and concentration measurement

Protein was isolated at 3 dpf. Zfl from six groups (F0-KO, Ctrl-Scr, UI_CRISPR_, MO-KD, Ctrl-MO, UI_MO_) were collected and decapitated. Then, 80–100 zfl heads per group were pooled in RIPA Lysis and Extraction Buffer (Thermo Fisher Scientific, Bremen, Germany) with 1× concentrated Protease inhibitor (Pierce Inhibitor Cocktail Tablets; Roche) (1 μl of RIPA buffer/zfl head) and frozen on dry ice. After the tissue was ruptured mechanically, the suspension was incubated for 2 h on a roller incubator at 4 °C and centrifuged at 13,000 rpm for 20 min at 4 °C. The supernatant was stored at –80 °C. The concentration of protein lysates was measured using the Pierce BCA Protein Assay Kit (Thermo Fisher Scientific).

### Peptide preparation for liquid chromatography mass spectrometry (LC-MS)

Protein solutions were processed using the SP3-approach [38]. Peptides (10 μg) were further desalted with C18 ZipTips (Merck Millipore, Darmstadt, Germany) to ensure the complete removal of beads.

### Directional measurement of Zfhx4 using LC-MS

All chemicals were obtained from Sigma (Taufkirchen, Germany) unless otherwise noted. Dried peptides were dissolved in 10 μl of 0.1% formic acid including retention standards. Peptide separation was performed on a Dionex Ultimate 3000 RSLC nano HPLC system (Dionex GmbH, Idstein, Germany) coupled to an Orbitrap Fusion Lumos mass spectrometer (Thermo Fisher Scientific). Peptides were injected onto a C18 analytical column (400 mm length, 100 μm inner diameter, ReproSil-Pur 120 C18-AQ, 3 μm).

The samples were analyzed first by a standard data-dependent acquisition (DDA) method. Peptides were separated using a linear gradient from 5% to 35% solvent B (90% acetonitrile, 0.1% FA) at 300 nl/min within 60 min. Scans were performed between 330 and 1600 m/z (standard gain control and injection time settings); ions were subjected to higher-energy collision-induced dissociation (HCD: 1.0 Da isolation, threshold intensity 25,000, collision energy 28%) and fragments were analyzed in the Orbitrap.

Fourteen specific peptides were selected for targeted analyses of Zfhx4, Zfhx3 (paralog of Zfhx4), and 60S ribosomal protein L4 (Rpl4; loading control) based on detection in DDA mode (see Table S1 for peptide sequences). Peptides were separated during a 120 min gradient. MS1 spectra were acquired every 3 s. Target ions were subjected to HCD fragmentation (1.6 Da window) and product ions were analyzed in the Orbitrap with a maximum injection time of 100 ms.

### LC-MS data analysis

Raw processing of DDA data and database searches were performed using Proteome Discoverer 2.5.0.400 (Thermo Fisher Scientific). Peptides were identified using Mascot server version 2.8.1 (Matrix Science Ltd., London, UK) against the UniProt reference proteome for *Danio rerio* (as of 04/12/23) and a collection of common contaminants [39]. For details, refer to the Supplementary Materials and Methods.

Data from targeted measurements were analyzed using Skyline [40]. Validation of MS2 spectra was aided by a spectral library created on the PROSIT server [41]. Protein quantification was performed at the MS2 level. Zfhx3 and Zfhx4 levels were normalized against the Rpl4 abundance in each sample.

### Cartilage staining

At 4 dpf, zfl were fixed and cartilage-stained with Alcian Blue according to Walker & Kimmel [42].

### Imaging and morphometric measurements of zfl

Zfl imaging and Meckel’s-palatoquadrate angle (M-PQ) [43] and ethmoidal plate and Meckel’s cartilage lengths were measured using a ZEISS Stemi 508 stereomicroscope with a Nikon DSFi2 camera and NIS Elements.

### Statistical analyses

Craniofacial phenotypes and Zfhx4 fold changes detected by LC-MS were compared among groups using two-way analysis of variance (ANOVA). Survival was compared using Kaplan-Meier survival curves. The M-PQ angle was compared using two-sided *t*-tests. GraphPad Prism 9 was used for analyses and to generate graphs. Values of *p* ≤ 0.05 were considered significant.

### Literature and database searches

A search for patients carrying *ZFHX4* variants was performed against PubMed using the search terms “ZFHX4” and “ZFHX4 orofacial cleft” and against the DECIPHER database for *ZFHX4*.

### Web resources

Please refer to Supplementary Materials and Methods.

## Results

### CNV calling and filtering

Among the 50 nsCL/P patients, XHMM (our primary calling algorithm) detected 1277 CNVs and 733 were retained after the elimination of common CNVs. Of those, 36 CNVs were not detected in the respective parents and were identified as de novo. Eleven CNVs were also called using either CoNIFER and/or EXCAVATOR2. Ultimately, after excluding CNVs that did not meet additional filtering criteria described in Fig. 1A IV-VI, a heterozygous 86 kb deletion affecting exons 4−11 of *ZFHX4* remained and its de novo status was confirmed (Fig. 1A–C). The CNV (or a comparable CNV with significant overlap) was absent from DGV.

### Additional variants identified by targeted-sequencing and clinical exome data

Post-collapsing, the coverage of *ZFHX4* was 587×. While no truncating variants were observed in the controls, targeted-sequencing revealed three truncating variants in patients, two frameshift variants leading to a premature stop, and a splice donor variant (Fig. 1D, Table 1). One of three truncating variants was identified as de novo. The segregation status of the other two variants could not be investigated owing to lack of parental DNA. The rare variant association test in the targeted-sequencing cohort revealed an association of nsCL/P with truncating variants in *ZFHX4* (*p* = 0.01575). In addition, a homozygous *ZFHX4* missense variant was identified by a clinical exome analysis of an individual with CL/P, microcephaly, and micrognathia born to unaffected parents carrying the variant in the heterozygous state (Table 1). This variant (NM_024721.4:c.1957T>C, NP_078997.4:Tyr653His) is located in a canonical zinc-finger motif of ZFHX4 which allowed to model its proposed conformation with high confidence [44] (Fig. 1E). Modeling of the zinc-finger structure showed that the hydroxyl group of the Tyr653 side chain could form a hydrogen bond with the imidazole ring of His662, which is part of the C2H2-type zinc-finger motif (Cys646, Cys649, His662, His667) that coordinates the metal ligand (Fig. 1F).

### Literature search of *ZFHX4* variants in individuals with orofacial clefting

PubMed and DECIPHER database searches revealed nine individuals [14, 45–48] with syndromic/nonsyndromic forms of orofacial clefts carrying *ZFHX4* variants or deletions affecting *ZFHX4* (Table 1).

### *Zfhx4/zfhx4* expression in mouse and zf during craniofacial development

**Fig. 2:**
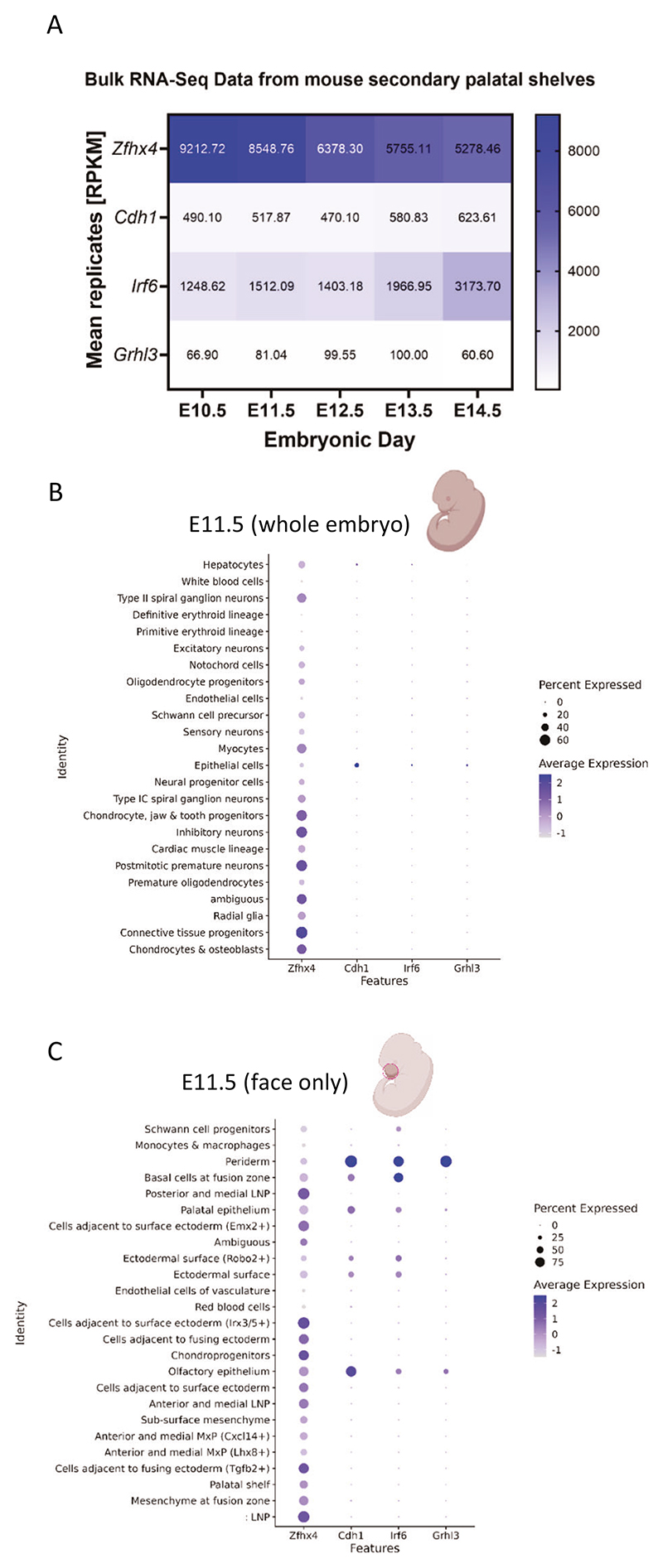
Expression pattern of *Zfhx4* in mouse embryos. **A** Heatmap of mean replicates in Reads Per Kilobase Million (RPKM) of *Zfhx4* and the established orofacial clefting genes (*Cdh1, Irf6*, and *Grhl3*) from bulk RNA-Seq data for mouse secondary palatal shelves (embryonic days E10.5–E14.5). Dot plots of gene expression from single-cell RNA-Seq data for the **B** whole mouse embryo and **C** mouse embryo face at E11.5. The color of the dots corresponds to the average scaled expression level. The size of the dots corresponds to the percentage of cells that expressed the gene in the respective cell type.

At the amino acid level we found 92% conservation between human and mouse ZFHX4 and 72% between human and zf (Fig. S1). Using published RNA-Seq datasets for mouse palatal shelves, expression levels of *Zfhx4* were higher than those of other OFC genes (*CDH1, IRF6*, and *GRHL3*) at all five timepoints examined (Fig. 2A, Fig. S2). *Grhl3* showed the lowest expression levels among the four genes (maximum value: *Grhl3*_E13.5_ = 100 RPKM).

*Zfhx4* was expressed in many cell types, especially in neuronal cells, chondrocytes, and connective tissue progenitors, based on scRNA-Seq data for whole mouse embryos at E11.5 (for more stages refer to Fig. S3). The other three OFC genes were mainly expressed in epithelial cells (Fig. 2B). ScRNA-Seq data from mouse face at E11.5 confirmed these findings. Whereas the established OFC genes were mainly expressed in peridermal/epithelial cells, *Zfhx4* showed a broad expression pattern (Fig. 2C). In situ hybridization and scRNA-Seq data from different timepoints of zf development supported these findings (Fig. S4–S7).

### zfhx4 F0-KO and MO-KD zfl present with craniofacial anomalies

**Fig. 3:**
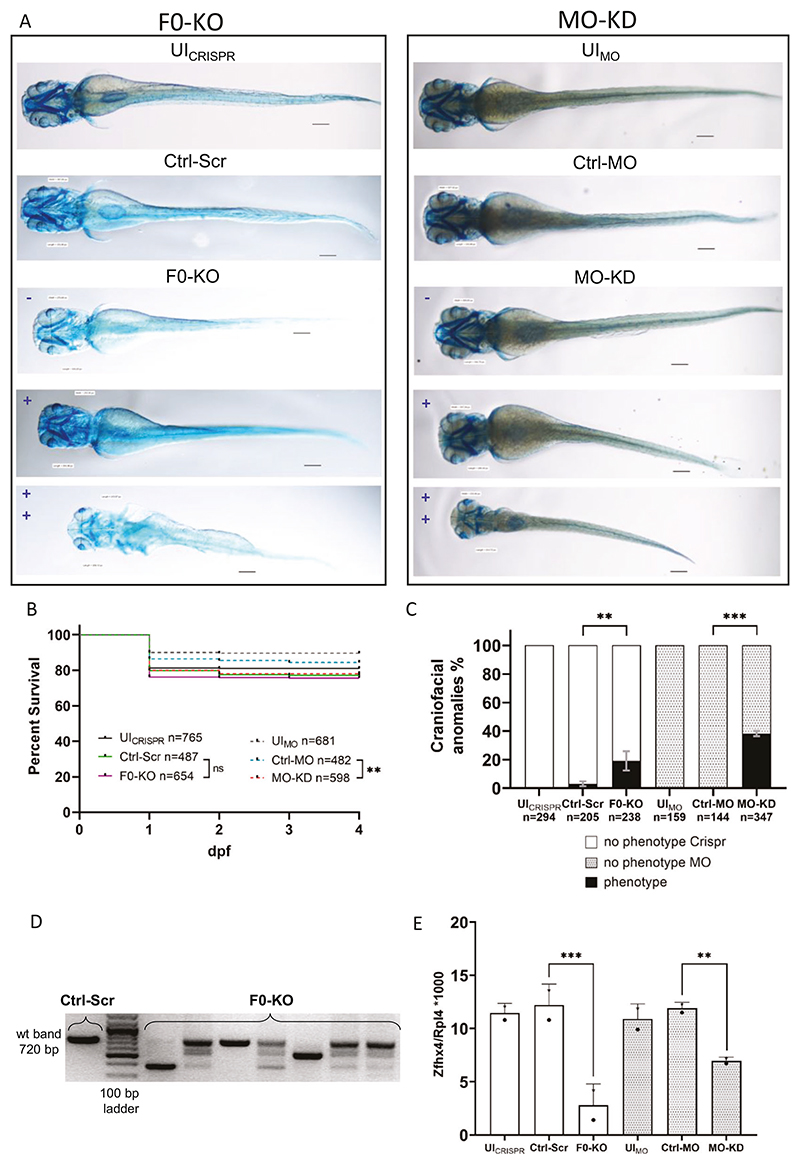
Effect of F0-generation knock-out (F0-KO) and translation-blocking morpholino knock-down (MO-KD) of *zfhx4* in zebrafish larvae (zfl). **A** Ventral view of whole-mount Alcian blue-stained zfl at 4 dpf. For F0-KO (left) and MO-KD (right), the spectrum of the phenotypes is shown in blue (– = no phenotype, += mild phenotype, ++ = strong phenotype). Scale bar = 113 μm. **B** Survival curves for each group (*N* = 3). Survival of F0-KO did not differ significantly from that of the Ctrl-Scr group, whereas survival was lower in MO-KD than in Ctrl-MO. Comparisons were performed using Mantel-Cox tests. Survival rates at 4 dpf: UI_CRISPR_: 81.1%, Ctrl-Scr: 77.2%, F0-KO: 75.7%, UI_MO_: 89.6%, Ctrl-MO: 84.4%, MO-KD: 78.1%. **C** Percentages of surviving zfl at 4 dpf with craniofacial anomalies (phenotype) and no phenotype in different groups (*N* = 3). No distinction between phenotype severities was made. F0-KO and MO-KD injection resulted in craniofacial anomalies in 19.13% and 38.17% of zfl, respectively, compared with rates of anomalies of 3.09% in Ctrl-Scr zfl and 0% in UI_CRISPR/MO_ and Ctrl-MO zfl. Comparisons were performed using two-way analysis of variance (ANOVA) and Tukey’s multiple comparisons tests. Data are presented as means with standard error of the mean (SEM). **D** PCR validation of the disruption of *zfhx4* exon 3 in F0-KO zfl at 4 dpf. Primer binding sites are located on adjacent introns. Expected wt band size, 720 bp. **E** Liquid chromatography mass spectrometry (LC-MS) analysis of protein lysates from heads of 3 dpf zfl (*N* = 2). Depicted are Zfhx4 fold changes relative to the 60S ribosomal protein L4 (Rpl4) abundance in each sample. Zfhx4 levels were decreased by 77.1% in F0-KO compared with levels in Ctrl-Scr and by 41.6% in MO-KD compared with levels in Ctrl-MO. Individual data for LC-MS run1 (stars) and run2 (dots) are plotted. Comparisons were performed using two-way analysis of variance (ANOVA) and Tukey’s multiple comparisons tests. Data are presented as means with standard deviation (SD). ns, *p* > 0.05, **p ≤ 0.01, ***p ≤ 0.001; dpf days post-fertilization, UI_CRISPR/MO_ uninjected wild-type zfl, Ctrl-MO Control MO-injected zfl, Ctrl-Scr Scrambled control-injected zfl, wt wild-type.

To assess the role of *zfhx4* in zfl development, we generated F0-KO and MO-KD and performed cartilage staining at 4 dpf. Controls (UI_CRISPR_, UI_MO_, Ctrl-Scr, and Ctrl-MO zfl) generally showed normal physiological development, including craniofacial development (Fig. 3A upper two panels). Zfl injected with the F0-KO mix and MO-KD resulted in anomalies with various degrees of severity and penetrance (Fig. 3A lower three panels, C). Survival rates did not differ significantly between F0-KO and Ctrl-Scr groups (*p* = 0.5417, Mantel-Cox test) but were slightly lower in MO-KD than in the Ctrl-MO group (*p* ≤ 0.01, Mantel-Cox test) (Fig. 3B). The penetrance of craniofacial anomalies was higher in the MO-KD group than in the F0-KO group (Fig. 3C). We observed relevant phenotypes, such as a reduced head size, shortened and misshaped jaw, and ethmoidal plate structures, in 19% of F0-KO zfl compared with 3% of Ctrl-Scr zfl (*p* ≤ 0.01, two-way ANOVA) and in 38% of MO-KD zfl compared with 0% of Ctrl-MO zfl (*p* ≤ 0.001, two-way ANOVA). The phenotypes described are similar to those seen in other studies disrupting OFC genes in zfl [49]. Zfl with a severe craniofacial phenotype may also present with reduced bodylength (Fig. 3A).

Genotyping revealed efficient mutagenesis of zfl (Fig. 3D). LC-MS analysis of protein lysates of zfl at 3 dpf demonstrated that Zfhx4 levels were 77.1% lower in F0-KO than in Ctrl-Scr zfl (*p* ≤ 0.001, two-way ANOVA) and 41.6% lower in MO-KD than in Ctrl-MO zfl (*p* ≤ 0.01, two-way ANOVA) (Fig. 3E), while no significant reduction in the Zfhx4 paralog Zfhx3 was detected (Fig. S8).

**Fig. 4:**
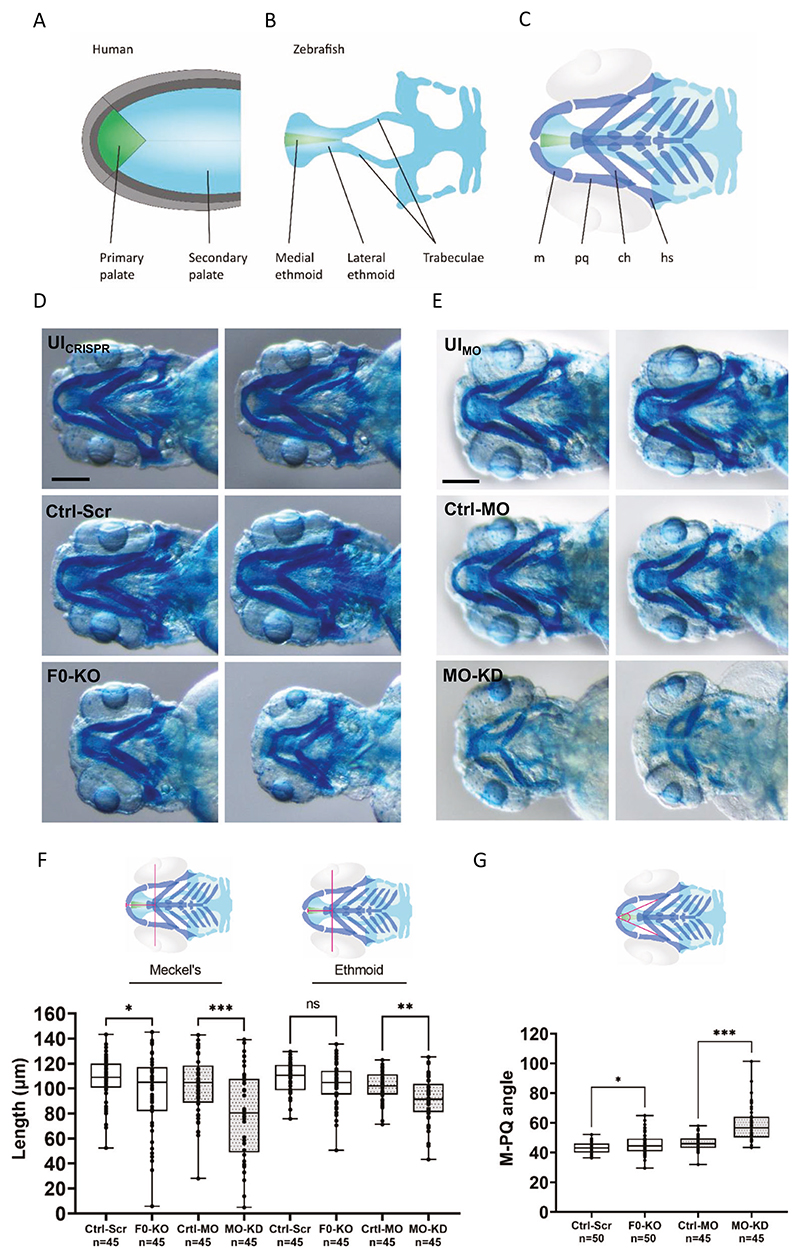
Craniofacial outcomes of *zfhx4* F0-generation knock-out (F0-KO) and translation-blocking morpholino knock-down (MO-KD) in zebrafish larvae (zfl). **A** Schematic overview of the human primary (green) and secondary palate (light blue) (ventral view). **B** Schematic overview of the zfl neurocranium with medial (green) and lateral ethmoid (light blue) (ventral view). Both structures are homologous structures to the human palate as indicated by the corresponding colors. **C** Ventral view of the zfl cartilage structures of the neurocranium (green and light blue) and viscerocranium (dark blue). The eyes are outlined in light gray. **D** Ventral view of the craniofacial region of whole-mount Alcian blue stained zfl at 4 dpf as represented schematically in C (for a different zfl than that shown in Fig. 3A). Comparison of craniofacial phenotypes between F0-KO zfl against controls (UI_CRISPR_ and Ctrl-Scr). Scale bar = 200 μm. **E** Ventral view of the craniofacial region of whole-mount Alcian blue-stained zfl at 4 dpf as represented schematically in C. Comparison of craniofacial phenotypes between MO-KD-injected zfl against controls (UI_MO_ and Ctrl-MO). Scale bar = 200 μm. **F** Length of Meckel’s cartilage and ethmoid plate measured as indicated by the pink lines of 4 dpf old, Alcian blue-stained F0-KO- and Ctrl-Scr-injected zfl (mean length Meckel’s: F0-KO: 96.9 μm, Ctrl-Scr: 107.7 μm, difference between means: 10.8 ± 5.4 μm; mean length ethmoid: F0-KO: 103.4 μm, Ctrl-Scr: 108.9 μm, difference between means 5.5 ± 3.1 μm) and MO-KD- and Ctrl-MO-injected zfl (mean length Meckel’s: MO-KD: 79.5 μm, Ctrl-MO: 102.9 μm, difference between means: 23.5 ± 6.3 μm; mean length ethmoid: MO-KD: 91.1 μm, Ctrl-MO: 101.5 μm, difference between means: 10.4 ± 3.2 μm). Comparisons were performed using unpaired, two-tailed Welch’s *t* tests. **G** Measurement of the Meckel’s-palatoquadrate (M-PQ) angle as represented by the pink angle, which is a reliable parameter to assess craniofacial outcomes as suggested by Raterman et al. [43]. Comparison of M-PQ angles in 4 dpf old, Alcian blue-stained F0-KO- and Ctrl-Scr-injected zfl (mean M-PQ angle: F0-KO: 45.6°, Ctrl-Scr: 43.1°, difference between means 2.5° ± 1.1°) and MO-KD- and Ctrl-MO-injected zfl (mean M-PQ angle: MO-KD: 58.8°, Ctrl-MO: 46.4°, difference between means 12.5° ± 2.0°). Comparisons were performed using unpaired, two-tailed Student’s *t* tests. ns, *p* > 0.05, *p ≤ 0.05, **p ≤ 0.01, ***p ≤ 0.001. m Meckel’s, pq Palatoquadrate, ch Ceratohyal, hs Hyosympletic, dpf days post-fertilization, UI_CRISPR/MO_ uninjected wild-type zfl, Ctrl-MO Control MO-injected zfl, Ctrl-Scr Scrambled controlinjected zfl.

To further evaluate the consequences of *zfhx4* disruption on craniofacial development, we specifically assessed the ethmoid (homolog of the human palate; Fig. 4A, B) and Meckel’s cartilage (Fig. 4C) in zfl at 4 dpf at higher magnification (Fig. 4D, E). There were no differences in parameter values among UI_CRISPR_, UI_MO_, Ctrl-Scr, and Ctrl-MO zfl. F0-KO and MO-KD zfl exhibited shorter Meckel’s cartilage and reduced ethmoid plate lengths (Fig. 4D, E).

We performed a quantitative analysis of Meckel’s cartilage and ethmoid plate lengths (Fig. 4F) and the M-PQ angle (Fig. 4G) using randomly chosen zfl within each group (without considering the presence or severity of craniofacial phenotypes). Meckel’s cartilage length, measured from the level of the interpupillary distance, was significantly shorter in F0-KO zfl (mean 96.9 μm) than in Ctrl-Scr zfl (mean 107.7 μm) (*p* ≤ 0.05, unpaired, two-tailed *t* test). A stronger effect was observed in MO-KD zfl (mean length: MO-KD: 79.5 μm, Ctrl-MO: 102.9 μm; *p* ≤ 0.001, unpaired, two-tailed *t* test) (Fig. 4F). The ethmoid plate length, measured from the level of the interpupillary distance, was significantly shorter in MO-KD zfl (mean 91.1 μm) than in Ctrl-MO zfl (mean 101.5 μm) (*p* ≤ 0.01, unpaired, two-tailed t test), but did not differ significantly between F0-KO (mean 103.4 μm) and Ctrl-Scr zfl (mean 108.9 μm) (*p* > 0.05, unpaired, two-tailed t test) (Fig. 4F). The M-PQ angle was significantly larger in F0-KO zfl (mean 45.6°) than in Ctrl-Scr zfl (mean 43.1°) (*p* ≤ 0.05, unpaired, two-tailed *t* test). The difference was greater between MO-KD (mean 58.8°) and Ctrl-MO zfl (mean 46.4°) (*p* ≤ 0.001, unpaired, two-tailed *t* test) (Fig. 4G).

## Discussion

We attempted to identify highly penetrant candidate genes for nsCL/P. ES data provided a valid basis for identifying CNVs, although very conservative filtering and validation are required. In particular, we identified a de novo heterozygous 86 kb deletion affecting major parts of *ZFHX4*, which encodes a transcription factor with 23 zinc finger motifs and four DNA-binding homeodomains (Fig. 1C) [50]. Targeted-sequencing of *ZFHX4* in our in-house nsCL/P and the AGORA cohort revealed three additional truncating variants.

To analyze the functions of *ZFHX4* in early craniofacial development, we established F0-KO and MO-KD zf models. *ZFHX4* was identified as suitable candidate for functional studies in zf, with only one orthologue (*zfhx4*) and high amino acid conservation (Fig. S1). *Zfhx4/zfhx4* was expressed in mouse/zf craniofacial tissues and cell lineages at time points relevant to craniofacial development (Fig. 2, Fig. S3–S7).

The disruption (F0-KO) and transient suppression (MO-KD) of *zfhx4* resulted in craniofacial changes in zfl at 4 dpf, albeit with differences in penetrance (Figs. 3, 4). These differences could be explained by the ability of MO-KD to bind maternal *zfhx4* RNA possibly present in the zf zygote. Since CRISPR acts at the DNA level, such maternal mRNA would not be affected by F0-KO and could lead to a rescue-like effect. However, two studies that analyzed the transcriptome of early zf zygotes, 2-cell stage –9 h post fertilization and 0.75–5.5 h post fertilization, respectively, with different methods, could not detect any significant expression of *zfhx4*, making the mentioned hypothesis obsolete [51, 52]. Nevertheless, the discrepancies in phenotype penetrance and severity between knockdowns and knockouts have been described and also assessed for some loci [53]. In our opinion, the most probable reason for the discrepancies in phenotype penetrance is the generation of hypomorphic alleles with F0-KO that still lead to functional protein or more complex compensatory mechanisms.

While the initial patient in this study presented with nsCL/P, individuals with *ZFHX4* variants or CNVs that disrupt *ZFHX4* exhibit both nonsyndromic and syndromic orofacial clefts, including nervous system abnormalities (e.g., developmental delay or intellectual disability), musculoskeletal or limb abnormalities, and craniofacial abnormalities in addition to the cleft such as microcephaly or micrognathia, with the latter being present in the majority of individuals with syndromic forms of clefting (Table 1). Regarding our zf models, the ethmoidal plate, which is structurally homologous to the human palate, was significantly shorter in MO-KD and slightly shorter in F0-KO, and Meckel’s cartilage was significantly shorter in both F0-KO and MO-KD (Fig. 4F). Of note, this may resemble micrognathia in humans [54]. The M-PQ angle, a well-established measure for craniofacial outcomes [43], was larger in F0-KO and MO-KD (Fig. 4G), indicative of micrognathia but also microcephaly [43]. These observations further support the causal role of the gene in craniofacial anomalies and a broader phenotypic spectrum (beyond nsCL/P). This was recently confirmed in a study by Nakamura et al., revealing that *Zfhx4-*deficient mice died within one day and presented with skeletal abnormalities and a cleft palate resulting from a failure in elevation and fusion of the palatal shelves [17]. Interestingly, mouse and zf orthologues of *CDH1, IRF6*, and *GRHL3*, associated with autosomal dominant nonsyndromic or syndromic forms of clefts with apparently nonsyndromic appearance, showed a narrow expression pattern during embryonic development, while mouse and zf orthologues of *ZFHX4* were much more widely expressed (Fig. 2, Figure S3–S7). This could explain, in part, why *ZFHX4* leads to a much wider phenotypic spectrum than just nsCL/P. DECIPHER also lists individuals with *ZFHX4* variants or CNVs that disrupt *ZFHX4* that have a syndrome without orofacial clefting (not included in Table 1), which may be explained by incomplete penetrance and modifying factors.

Collectively, the data compiled in this study demonstrate that different types of variants, from small missense and truncating variants in *ZFHX4* to larger CNVs affecting only *ZFHX4* or additional genes, either inherited or acquired de novo, could lead to OFC. The type of cleft caused by *ZFHX4* variants or CNVs affecting *ZFHX4* is variable, including CL/P and CPO, either nonsyndromic or syndromic. Currently, it appears that large deletions are more likely to result in CPO or a highly arched palate. Of note, there is evidence that CPO and a highly arched palate share genetic risk factors [55]. By contrast, small variants may be more likely to cause CL/P than CPO. Of note, the fact that genetic testing is more likely to be performed for individuals with complex syndromes than individuals with apparently nonsyndromic forms of OFC may lead to a reporting bias towards syndromic forms of OFC.

Regarding the postulated mode of inheritance, all individuals from the literature and DECIPHER, and four of five of the newly presented individuals, had heterozygous variants or CNVs affecting *ZFHX4*. With a pLI score of 1, *ZFHX4* is highly constrained, suggesting that the loss of substantial portions of one allele results in haploinsufficiency. However, one individual presented in this study carried a novel homozygous missense variant in *ZFHX4*, whereas her parents were heterozygous carriers with no malformations or abnormalities (Table 1, Supplementary Material). This is the first study to present an individual with an apparent recessive inheritance of *ZFHX4* variants. The structural modelling of ZFHX4 showed that the respective amino acid change Tyr653His might disrupt the formation of a hydrogen bond with the imidazole ring of His662, which is part of the C2H2-type zinc-finger motif (Fig. 1F). This disruption might alter the structural conformation as well as the ability for zinc-coordination in this part of the protein, both of which could be important for the ability of the transcription factor ZFHX4 to bind DNA. This observation raises the possibility that, in addition to heterozygous *ZFHX4* variants, homozygous or biallelic *ZFHX4* variants might also contribute to OFC risk.

In conclusion, our results support the role of *ZFHX4* in OFC. Variants in *ZFHX4* or CNVs can lead to both typical subtypes of OFC − CL/P and CPO. Also, *ZFHX4* disruption can lead to both nonsyndromic and syndromic OFC. Based on our findings and recent studies, *ZFHX4* variants are associated with an extremely broad phenotypic spectrum, from complex malformation syndromes to nonsyndromic isolated malformations. Accordingly, more data are needed to systematically assess genotype–phenotype relationships and to identify modifying factors. *ZFHX4* should be considered a causative factor not only in patients with severe complex syndromes but also in patients with mild syndromic and even nonsyndromic OFC.

## Supplementary Material

**Supplementary information** The online version contains supplementary material available at https://doi.org/10.1038/s41431-024-01775-9.

Supplementarymaterial and figures

Supplementary figures

Table S1. Primer, morpholino, sgRNA and peptide sequences.

## Figures and Tables

**Table 1:** Genetic evidence for the role of *ZFHX4* in orofacial clefting.

Source	Mutation type	Affected exon *ZFHX4*	Nucleotide change (NM_024721.4) ^a^	Amino acid change (NP_078997.4)	Zygosity	De novo (y/n)	Size	Syndromic (y/n)	OFC phenotype	Micrognathia (y/n)
Present study	CNV, In- frame deletion	4–11	c.3105_10835del	p.(Lys1035_Asp3612delinsAsn)	HET	y	86,33 kb	n	CL/P	n
Present study (Zametica)	Frameshift deletion	2	c.1863_1864del	p.(Leu622GlyfsTer26)	HET	na	2 bp	n	CL/P	Retrognathia
Present study (Zametica)	SNV, splice donor	Intron 9	c.3964+2T>A	p.?	HET	na	1 bp	n	CL/P	n
Present study (Zametica)	SNV, frameshift deletion	2	c.1927del	p.(Thr643ProfsTer21)	HET	y	1 bp	n	CL/P	n
Present study (Girisha)	SNV, missense	2	C.1957T > C	p.(Tyr653His)	HOM	n	1 bp	y	CL/P	y
Palomares et al. [45]	Deletion, start loss		C.−3231464_*6395221 del	p.Metl?	HET	y	9.80 Mb	y	Highly arched palate	y
Palomares et al. [45]	Deletion, start loss		C.−2993631 _*2514645del	p.Metl?	HET	y	5.69 Mb	y	Highly arched palate	y
Palomares et al. [45]	Deletion, start loss		c.−495025_7082del	p.Metl?	HET	n	0.66 Mb	y	Cp^b^	n
Bishop et al. [14]	SNV, frameshift deletion	10	c.5595del	p.(Glu 1866LysfsTer8)	HET	y	1 bp	n	CL/P	Not known
Bishop et al. [14]	SNV, stopgain	10	c.7990 C > T	p.(Gln2664Ter)	HET	y	1 bp	n	CL/P	Not known
Fontana et al. [46]	SNV, splice donor	Intron 3	c.3093+1 G > T	p.?	HET	y	1 bp	y	Highly arched palate	y
Créton et al. [47]	SNV, frameshift deletion	2	c.2513del	p.(Asn838ThrfsTer7)	HET	y	1 bp	y	CL/P	y^c^
Sorrentino et al. [48]	Frameshift deletion- insertion	9	c.3943_3945delinsAA	p.(Glu1315LysfsTer29)	HET	y	2 bp	n^d^	CL/P	n
DECIPHER vi 1.13, Patient 412653	SNV, missense	10	c.8882 T > C	p.(Met2961Thr)	HET	y	1 bp	y	CP	n
DECIPHER vi 1.13, Patient 284919	SNV, stopgain	2	c.2041 C>T	p.(Gln681Ter)	HET	n^e^	1 bp	y	CL/P	n
DECIPHER vi 1.13, Patient 268410	SNV, missense	5	c.3326A > T	p.(Glu1109Val)	HET	n	1 bp	y	Highly arched palate	y

*na* not available, *y* yes, *n* no.

a All variants are absent from GnomAD v2.1.1.

b Individual from Belligni et al. 2010. In this study, four additional family members with syndromal phenotypes are reported, including CPO (maternal grandmother and sister of maternal grandmother) and nasal voice (mother and maternal great-grandmother).

c Also in the mother without a *ZFHX4* mutation.

d The pregnancy was terminated after an orofacial cleft was detected on ultrasound examination. The status was apparently nonsyndromic. Postmortem examination of the fetus confirmed the absence of other major congenital anomalies. However, other syndromic features that could not be assessed postmortem, such as developmental delay or mental retardation, could not be completely excluded.

e Inherited from the mother with CL/P (unilateral cleft lip).

## Data Availability

The data that support the findings of this study are available on request from the corresponding authors. The data are not publicly available due to privacy or ethical restrictions.
