# Supplementary material for: Role of ZFHX4 in orofacial clefting based on human genetic data and zebrafish models
Source: Eur J Hum Genet. 2024 Dec 19;33(5):595–606. doi: 10.1038/s41431-024-01775-9 (PMC7617551; doi:10.1038/s41431-024-01775-9)
Supplement: Supplementary file 1 — Supplementary Material and Methods [file 41431_2024_1775_MOESM1_ESM.pdf]

Supplementary Materials & Methods

Role of *ZFHX4* in orofacial clefting based on human genetic data and zebrafish models

Nina Ishorst[*,‡] (1,2), Selina Hölzel[*] (1,3), Carola Greve (1,#), Öznur Yilmaz (2), Tobias Lindenberg (2), Jessica Lambertz (2), Dmitriy Drichel (4), Berina Zametica (1), Enrico Mingardo (3), Jeshurun C. Kalanithy (1,2), Khadija Channab (3), Duygu Baydemir (1,2), Sabrina Henne (1), Franziska Degenhardt (1,†), Anna Siewert (1), Michael Dixon (5), Teresa Kruse (6), Edwin Ongkosuwito (7), Katta M. Girisha (8), Shruti Pande (8), Stefanie Nowak (1), Gregor Hagelueken (9), Matthias Geyer (9), Carine Carels (10), Iris A.L.M. van Rooij (11), Kerstin U. Ludwig (1), Benjamin Odermatt[**,‡] (2,3), Elisabeth Mangold[**,‡] (1)

Affiliations:

1 Institute of Human Genetics, University of Bonn, School of Medicine & University Hospital Bonn, Bonn, Germany

2 Institute of Anatomy, Division of Neuroanatomy, University of Bonn, School of Medicine & University Hospital Bonn, Bonn, Germany

3 Institute of Anatomy and Cell Biology, University of Bonn, School of Medicine & University Hospital Bonn, Bonn, Germany

4 Cologne Center for Genomics, University of Cologne, Cologne, Germany

5 Faculty of Biology, Medicine & Health, University of Manchester, Manchester M13 9PL, UK

6 University of Cologne, Faculty of Medicine and University Hospital Cologne, Department of Orthodontics, Cologne, Germany

7 Department of Dentistry, Section of Orthodontics and Craniofacial Biology, Radboud Institute for Health Sciences, Radboud University Medical Center, Nijmegen, The Netherlands

8 Department of Medical Genetics, Kasturba Medical College, Manipal, Manipal Academy of Higher Education, Manipal, India

9 Institute of Structural Biology, University of Bonn, Bonn, Germany

10 Department of Human Genetics, KU Leuven, Leuven, Belgium

11 Department for Health Evidence, Radboud University Medical Center, Nijmegen, The Netherlands

# Current address: LOEWE Centre for Translational Biodiversity Genomics, Frankfurt am Main, Germany

† Current address: Department of Child and Adolescent Psychiatry, Psychosomatics and Psychotherapy, University Hospital Essen, University of Duisburg-Essen, Duisburg, Germany

[*] First authors contributed equally.

[**] Last authors contributed equally.

[‡]Corresponding authors:

Nina Ishorst: nina.ishorst@uni-bonn.de

Elisabeth Mangold: e.mangold@uni-bonn.de

Benjamin Odermatt: b.odermatt@uni-bonn.de

Clinical summary of individual with syndromic orofacial clefting and recessive inheritance

We ascertained a six-years-old female born to a consanguineously married couple. There is no history of any significant events during the antenatal period and at the time of birth. She presented with cleft of lip and palate, for which she was operated at seven months of age. Her developmental milestones were age appropriate. On examination at six years of age, her head circumference was 48 cm (-3.2 SD), weight was16.8 kg (-1.6 SD) and height was 106 cm (-1.8 SD). She has a transverse palmar crease in left hand, sacral dimple, upslanting palpebral fissures, broad nasal root and nasal bridge, mildly dysmorphic ears with lobule directly attached to head without normal gap. Her intellectual functioning is apparently normal (IQ testing is not done).

Family history: Her younger sibling expired at 45 days of life. She had a clinical diagnosis of Apert syndrome, though it was not confirmed by molecular testing.

Detailed methods for Directional measurement of Zfhx4 using Liquid Chromatography Mass Spectrometry (LC-MS)

All chemicals from Sigma (Taufkirchen, Germany) unless otherwise noted.

Peptide preparation

Protein solutions were processed with the SP3-approach [1]. Briefly, protein lysate with 50 µg protein were subjected to cysteine reduction and alkylation with 20 mM DTT and 40 mM acrylamide in 50 mM triethylammonium bicarbonate (TEAB). Then a mixture of hydrophilic carboxylate-coated magnetic beads (equal amounts of Sera-Mag SpeedBeads, GE Healthcare, cat. no. 45152105050250, and cat. no. 65152105050250) were added at a bead:protein ratio of 10:1 (w/w). Protein binding was induced by adding 3 volumes of ethanol and subsequent mixing for 5 min. Beads with bound protein were then washed three times

with 80 % ethanol and finally subjected to overnight tryptic digestion at 37 °C using a trypsin:protein ratio of 1:25. Peptide solutions were separated from the magnetic beads, dried in a vacuum concentrator, and stored at -20 °C. Before measurements, 10 µg of peptides were further desalted with C18 ZipTips (Merck Millipore, Darmstadt, Germany) to ensure complete removal of beads.

LC-MS analysis

Dried peptides were dissolved in 10 µl 0.1 % formic acid (solvent A) including retention time standard peptides. Peptide separation was performed on a Dionex Ultimate 3000 RSLC nano HPLC system (Dionex GmbH, Idstein, Germany) coupled to an Orbitrap Fusion Lumos mass spectrometer (Thermo Fisher Scientific, Bremen, Germany). The autosampler was operated in µl-pickup mode. For each sample, 15 % were injected onto a C18 analytical column (400 mm length, 100 µm inner diameter, ReproSil-Pur 120 C18-AQ, 3 µm).

The samples were analyzed first by a standard data-dependent (DDA) method. Peptides were separated during a linear gradient from 5 % to 35 % solvent B (90 % acetonitrile, 0.1 % FA) at 300 nl/min within 60 min. Data-dependent acquisition was performed on ions between 330 and 1600 m/z scanned in the Orbitrap detector every 3 seconds (R = 60,000, standard gain control and inject time settings). Polysiloxane (*m/z* 445.12002) was used for internal calibration. Z > 1 ions were subjected to higher-energy collision induced dissociation (HCD: 1.0 Da quadrupole isolation, threshold intensity 25,000, collision energy 28%) and fragments analyzed in the Orbitrap (R = 15,000). Fragmented precursor ions were excluded from repeated analysis for 20 s.

In total 14 specific peptides were selected for targeted analysis based on detectability in DDA measurements: Zfhx4: GSASLASSADQSPR, FSSDSLEALSGHVATER, HQQSEGLR, ETLGIATAGK, VQETLGNQVDR, Zfhx3: CEVCDYETNVAR, IHMTSEK, FTDYQLR, Rpl4:

SGQGAFGNMCR, NIPGITLQSVSR, LAPGGHIGR, LDDLYGTWR, VDYNLPMHK, MSITDLNR.

Peptides were separated during a 120 min gradient. MS1 spectra were acquired from 330 to 1600 m/z every 3 seconds (R = 120,000) Target ions were subjected to HCD fragmentation (1.6 Da window), and product ions analyzed in the Orbitrap (R = 15,000) with gain control target 60,000 and maximum inject time 100 ms.

Data analysis

Raw data processing of DDA data and analysis of database searches were performed with Proteome Discoverer software 2.5.0.400 (Thermo Fisher Scientific). Peptide identification was done with an in-house Mascot server version 2.8.1 (Matrix Science Ltd, London, UK) against the Uniprot reference proteome for Danio rerio (as of 04/12/23) and a collection of common contaminants [2]. Precursor ion *m/z* tolerance was 10 ppm, fragment ion tolerance 20 ppm. Tryptic peptides (Trypsin/P) with up to two missed cleavages were searched, propionamide was set as a static modification of cysteines, while oxidation of methionine and acetylation of protein N-termini were set as dynamic modifications. Spectrum confidence of Mascot results was assessed by the Percolator algorithm 3.05 as implemented in Proteome Discoverer [3]. Spectra without high confident matches (q-value > 0.01) were sent to a second round Mascot search with semi-specific enzyme cleavage and changing the modification of cysteines with propionamide to dynamic.

Data from targeted measurements were analyzed in Skyline [4]. Validation of MS2 spectra was aided by a spectral library created on the PROSIT server [5]. Protein quantification was done on MS2 level. Zfhx3 and Zfhx4 levels were normalized on Rpl4 abundance in each sample.

Web Resources

AnnotSV web browser: https://lbgi.fr/AnnotSV/runjob

BioStudies: https://www.ebi.ac.uk/biostudies/

BWA-MEM: https://github.com/lh3/bwa

CADD: https://cadd.gs.washington.edu/

Clustal Omega: https://www.ebi.ac.uk/Tools/msa/clustalo/

DECIPHER: https://www.deciphergenomics.org/

gnomAD: https://gnomad.broadinstitute.org/

MIPgen: https://github.com/shendurelab/MIPGEN/

PLINK/Seq: https://zzz.bwh.harvard.edu/plinkseq/

Primer3web: http://primer3.ut.ee/

Samtools: http://www.htslib.org/

UCSC Genome Browser (GRCh37/hg19): https://genome.ucsc.edu

VEP: https://www.ensembl.org/info/docs/tools/vep/index.html

ZEBRAHUB: https://zebrahub.ds.czbiohub.org/transcriptomics

Citations

1 Hughes CS, Moggridge S, Müller T, Sorensen PH, Morin GB, Krijgsveld J. Single-pot, solid-phase-enhanced sample preparation for proteomics experiments. *Nat Protoc* 2019; **14**: 68–85.

2 Frankenfield AM, Ni J, Ahmed M, Hao L. Protein Contaminants Matter: Building Universal Protein Contaminant Libraries for DDA and DIA Proteomics. *J Proteome Res* 2022; **21**: 2104–2113.

3 Käll L, Storey JD, MacCoss MJ, Noble WS. Assigning significance to peptides identified by tandem mass spectrometry using decoy databases. *J Proteome Res* 2008; **7**: 29–34.

4 Pino LK, Searle BC, Bollinger JG, Nunn B, MacLean B, MacCoss MJ. The Skyline ecosystem: Informatics for quantitative mass spectrometry proteomics. *Mass Spectrom Rev* 2020; **39**: 229–244.

5 Gessulat S, Schmidt T, Zolg DP, Samaras P, Schnatbaum K, Zerweck J *et al.* Prosit: proteome-wide prediction of peptide tandem mass spectra by deep learning. *Nat Methods* 2019; **16**: 509–518.
