# Supplementary material for: Role of ZFHX4 in orofacial clefting based on human genetic data and zebrafish models
Source: Eur J Hum Genet. 2024 Dec 19;33(5):595–606. doi: 10.1038/s41431-024-01775-9 (PMC7617551; doi:10.1038/s41431-024-01775-9)
Supplement: Supplementary file 2 — Supplementary Figures [file 41431_2024_1775_MOESM2_ESM.pdf]

Supplementary Figures

Role of *ZFHX4* in orofacial clefting based on human genetic data and zebrafish models

Nina Ishorst[*,‡] (1,2), Selina Hölzel[*] (1,3), Carola Greve (1,#), Öznur Yilmaz (2), Tobias Lindenberg (2), Jessica Lambertz (2), Dmitriy Drichel (4), Berina Zametica (1), Enrico Mingardo (3), Jeshurun C. Kalanithy (1,2), Khadija Channab (3), Duygu Baydemir (1,2), Sabrina Henne (1), Franziska Degenhardt (1,†), Anna Siewert (1), Michael Dixon (5), Teresa Kruse (6), Edwin Ongkosuwito (7), Katta M. Girisha (8), Shruti Pande (8), Stefanie Nowak (1), Gregor Hagelueken (9), Matthias Geyer (9), Carine Carels (10), Iris A.L.M. van Rooij (11), Kerstin U. Ludwig (1), Benjamin Odermatt[**,‡] (2,3), Elisabeth Mangold[**,‡] (1)

Affiliations:

1 Institute of Human Genetics, University of Bonn, School of Medicine & University Hospital Bonn, Bonn, Germany

2 Institute of Anatomy, Division of Neuroanatomy, University of Bonn, School of Medicine & University Hospital Bonn, Bonn, Germany

3 Institute of Anatomy and Cell Biology, University of Bonn, School of Medicine & University Hospital Bonn, Bonn, Germany

4 Cologne Center for Genomics, University of Cologne, Cologne, Germany

5 Faculty of Biology, Medicine & Health, University of Manchester, Manchester M13 9PL, UK

6 University of Cologne, Faculty of Medicine and University Hospital Cologne, Department of Orthodontics, Cologne, Germany

7 Department of Dentistry, Section of Orthodontics and Craniofacial Biology, Radboud Institute for Health Sciences, Radboud University Medical Center, Nijmegen, The Netherlands

8 Department of Medical Genetics, Kasturba Medical College, Manipal, Manipal Academy of Higher Education, Manipal, India

9 Institute of Structural Biology, University of Bonn, Bonn, Germany

10 Department of Human Genetics, KU Leuven, Leuven, Belgium

11 Department for Health Evidence, Radboud University Medical Center, Nijmegen, The Netherlands

# Current address: LOEWE Centre for Translational Biodiversity Genomics, Frankfurt am Main, Germany

† Current address: Department of Child and Adolescent Psychiatry, Psychosomatics and Psychotherapy, University Hospital Essen, University of Duisburg-Essen, Duisburg, Germany

[*] First authors contributed equally.

[**] Last authors contributed equally.

[‡]Corresponding authors:

Nina Ishorst: nina.ishorst@uni-bonn.de

Elisabeth Mangold: e.mangold@uni-bonn.de

Benjamin Odermatt: b.odermatt@uni-bonn.de

A Percent Identity Matrix - created by Clustal2.1

	hs_ZFHX4 (Q86UP3-5)	mm_ZFHX4 (H3BLK8)	dr_Zfhx4 (F6NXU9)
hs_ZFHX4 (Q86UP3-5)	100.00	92.17	72.23
mm_ZFHX4 (H3BLK8)	92.17	100.00	71.11
dr_Zfhx4 (F6NXU9)	72.23	71.11	100.00

B CLUSTAL O(1.2.4) multiple sequence alignment

```
hs_ZFHX4            METCDSPPISRQENGQSTSKLCGTTQLDNEVPEKVAGMEPDRENSSTDDNLKTDERKSEA   60
mm_ZFHX4            METCDSPPISRQENGQSTSKLCGMTQLDNEVPEKVAGIEPDRENSSSHDNLKTDERKSEV   60
dr_Zfhx4            METCDSPSLLRQENGQQISKLRETTHLDNEVPEKVPGMEPDKENSPADDNLRTEESLREI   60
                    ******* : ******. ***   *:********* *:***:*** :.***:*:*   *

hs_ZFHX4            LLGFSVENAAATQVTSAKEIPCNECATSFPSLQKYMEHHCPNARLPVLKDDNESEISELE   120
mm_ZFHX4            LLGFSIENAAATQVTSAKEIPCNECATSFPSLQKYMEHHCPNARLPVLKDD-ESETSELE   119
dr_Zfhx4            ASGLSAENAIG---TATKEIPCNECATSFSSLQKYMEHHCPNARLPLLKDENESEVSDLE   117
                      *:* *** .   *::************ ****************:***: *** *:**

hs_ZFHX4            DSDVENLTGEIVYQPDGSAYIIEDSKESGQNAQTGANSKLFSTAMFLDSLASAGEKSDQS   180
mm_ZFHX4            DSDVENLTGEIVYQPDGSAYIIEDSKESGQNAQTGANSKLFSTAMFLDSLASAGEKSDQS   179
dr_Zfhx4            DSDVENLTGEIVYQPDGSAFILEDSKEGGQNAQSGVK-SLFSPALFSNSQTAAGDKTEQS   176
                    *******************:*:*****.*****:*.: .*** *:* :* ::**:*::**

hs_ZFHX4            ASAPMSFYPQIINTFHIASSLGKPFTADQAFPNTSALAGVGPVLHSFRVYDLRHKREKDY   240
mm_ZFHX4            STAPVSFYPQIINTFHIASSLGKPFTADPAFPNTSALAGVGPVLHSFRVYDLRHKREKDY   239
dr_Zfhx4            ATAPMSFYPQVINTFHIASSLGKPFTADQAFPNTSALAGEGPVLHSFRVYDLRHKSDKDY   236
                    ::**:*****:***************** ********** *************** :***

hs_ZFHX4            LTSDGSAKNSCVSKDVPNNVDLSKFDGCVSDGKRKPVLMCFLCKLSFGYIRSFVTHAVHD   300
mm_ZFHX4            LTSDGSAKNSCVSKDVPNNVDLSKFDGCVSDGKRKPVLMCFLCKLSFGYIRSFVTHAVHD   299
dr_Zfhx4            LTIDGAAKNSCVSKDVPNNVDLSKFDGCISDGKRKPILMCFLCKLSFGYSRSFVTHAVHD   296
                    ** **:**********************:*******:************ **********

hs_ZFHX4            HRMTLNDEEQKLLSNKCVSAIIQGIGKDKEPLISFLEPKKSTSVYPHFSTTNLIGPDPTF   360
mm_ZFHX4            HRMTLNDEEQRLLSNKCVSAIIQGIGKDKEPLISFLEPKKSTSVYPNFSTTNLIGPDPTF   359
dr_Zfhx4            HRMTLNEQEQKLLSNKYVSAIIQGIGKDKEPLISFLEPKKNNSMLPHFSTANFMGPDPGI   356
                    ******::**:***** ***********************..*: *:***:*::**** :

hs_ZFHX4            RGLWSAFHVENGDSLPAGFAFLKGSASTSSSAEQPLGITQMPKAEVNLGGLSSLVVNTPI   420
mm_ZFHX4            RGLWSAFHVENGDSLQAGFAFLKGSASPSSSAEQPLGITHMPKAEVNLGGLSSLVVNTPI   419
dr_Zfhx4            RSLWNTFHMENGDSLQAGFAFLKGSASLASSADQSPRSAQMPKAELNLGVMATSALKSPV   416
                    *.**.:**:****** *********** :***:*    ::*****:*** ::: .:::*:

hs_ZFHX4            TSVSLSHSSSESSKMSESKDQENNCERPKESNVLHPNGECPVKSEPTEPGDEDEEDAYSN   480
mm_ZFHX4            TSVSLSHLSSESSKMSESKDQENNCERPKESTILHPNVGCPVKSEPTEPGDEDEEDAYSN   479
dr_Zfhx4            SSVSLQRSSPG----AGYRDQESNCERQKDLSTLHANGELPIKSEPRDVADAEEEDMYSN   472
                    :****.: *      :  :***.**** *: . ** *   *:**** : .* :*** ***

hs_ZFHX4            ELDDEEVLGELTDSIGNKDFPLLNQSISPLSSSVLKFIEKGTSSSSATVSDDTEKKKQTA   540
mm_ZFHX4            ELDDEEVLGELTDSIGNKDFPLLNQSISPLSSSVLKFIEKGTSSSSGTIAEDTEKKKQAA   539
dr_Zfhx4            ELDDDGV-CELEDSTSSKDFPLLNQSISPLSSSVLKFTERGTTSSVTV-ANDFEKTKHAA   530
                    ****: *  ** ** ..******************** *:**:**  . ::* **.*::*

hs_ZFHX4            -AVRASGSVASNYGISGKDFADASASKDSATAAHPSEIARGDEDSSATPHQHGFTPSTPG   599
mm_ZFHX4            AAGRSNGNVTNSYSIGGKDFADGSISRDGTTA-APSETTHGDEDS-STTHQHGFTPSTPG   597
dr_Zfhx4            ANLDYSNDSTS---GGSKDSCDSISGRGGSPPSLPLDLMRRDDESPGPLHQHAATPSTPG   587
                         ... :.    ..** .*.  .:..:    * :  : *::* .  ***. ******

hs_ZFHX4            TPGPGGDGSPGSGIECPKCDTVLGSSRSLGGHMTMMHSRNSCKTLKCPKCNWHYKYQQTL   659
mm_ZFHX4            TPGPGGDGSPGNGIECPKCDTVLGSSRSLGGHMTMMHSRNSCKTLKCPKCNWHYKYQQTL   657
dr_Zfhx4            TPGPG-EGSPGSGIECPKCDTVLGSSRSLGGHMTMMHSRNSCKTLKCPKCNWHYKYQQTL   646
                    ***** :****.************************************************

hs_ZFHX4            EAHMKEKHPEPGGSCVYCKTGQPHPRLARGESYTCGYKPFRCEVCNYSTTTKGNLSIHMQ   719
mm_ZFHX4            EAHMKEKHPEPGGSCVYCKTGQPHPRLARGESYTCGYKPFRCEVCNYSTTTKGNLSIHMQ   717
dr_Zfhx4            DAHMKEKHPESGGSCVYCRTGQPHPRLARGESYTCGYKPFRCEVCNYSTTTKGNLSIHMQ   706
                    :********* *******:*****************************************

hs_ZFHX4            SDKHLNNVQNLQNGNGEQVFGHSAPAPNTSLSGCGTPSPSKPKQKPTWRCEVCDYETNVA   779
mm_ZFHX4            SDKHLNNVQNLQNGNGEQVFGHSAPTPNTSLSGCGTPSPSKPKQKPTWRCEVCDYETNVA   777
dr_Zfhx4            SDKHLNNVQTLQNGGTEAIYNHTTPVTNTSLTGCGTPSPSKPKQKPTWRCEVCDYETNVA   766
                    *********.****. * ::.*::*. ****:****************************

hs_ZFHX4            RNLRIHMTSEKHMHNMMLLQQNMKQIQHNLHLGLAPAEAELYQYYLAQNIGLTGMKLENP   839
mm_ZFHX4            RNLRIHMTSEKHMHNMMLLQQNMKQIQHNLHLGLAPAEAELYQYYLAQNIGLTGMKLENP   837
dr_Zfhx4            RNLRIHMTSEKHMHNMMLLQQNMKQIQHNLHLGLAPAEAELYQYYLAQNIGLTGMKLENP   826
                    ************************************************************

hs_ZFHX4            ADPQLMINPFQLDPATAAALAPGLVNNELPPEIRLASGQLMGDDLSLLTAGELSPYISDP   899
mm_ZFHX4            AETQLLLNPFQFDSATAAALAPGLVNNELPPEIRLASGQLMGDDLSLLTAGELSPYISDP   897
```

Family BN00844
p.Leu622GlyfsTer26

Family 27
p.Thr643ProfsTer21

Girisha & colleagues
p.Tyr653His

```
dr_Zfhx4      SDPQMMINPFQLDPSTAAALTPGHVNNELPAELRLASGQLMGDDLSVLSAGELSPCINDP  886
              :: *:::****:* :*****:** ****** *:************:*:****** *.**

hs_ZFHX4      ALKLFQCAVCNKFTSDSLEALSVHVSSERSLPEEEWRAVIGDIYQCKLCNYNTQLKANFQ  959
mm_ZFHX4      ALKLFQCAVCNKFTSDSLEALSVHVNSERSLPEEEWRAVIGDIYQCKLCNYNTQLKANFQ  957
dr_Zfhx4      LLKLFQCAVCNKFSSDSLEALSGHVATERSLPEEEWRAVMGDVYQCKLCNYNTQLKANFQ  946
               ************:******** ** :************:**:*****************

hs_ZFHX4      LHCKTDKHMQKYQLVAHIKEGGKSNEWRLKCIAIGNPVHLKCNACDYYTNSVDKLRLHTT  1019
mm_ZFHX4      LHCKTDKHMQKYQLVAHIKEGGKSNEWRLKCIAIGNPVHLKCNACDYYTNSVDKLRLHTT  1017
dr_Zfhx4      LHCKTDKHMQKYQLVAHIKEGGKANQWRLKCIAIGNPVHLKCNACDYYSNSVEKLRLHAT  1006
              ***********************:*:*********************:***:*****:*

hs_ZFHX4      NHRHEAALKLYKHLQKQEGAVNPESCYYYCAVCDYTTKVKLNLVQHVRSVKHQQTEGLRK  1079
mm_ZFHX4      NHRHEAALKLYKHLQKQEGAVNSESCYYYCAVCDYSSKIKLNLVQHVRSVKHQQTEGLRK  1077
dr_Zfhx4      NQRHEAALKVYKHLQKQESACNSESCYYYCALCDYSSRAKLNLLQHLRSVKHQQSEGLRK  1066
              *:*******:********.* * ********:***::: ****:**:*******:*****

hs_ZFHX4      LQLHQQGLAPEEDNLSEIFFVKDCPPNELETASLGARTCDDDLTEQQLRSTSEEQSEEAE  1139
mm_ZFHX4      LQLHQQGLPSEEDNLSEIFFVKECPANELETASLGARNGEDELIEQQLKAASEEPSEDAG  1137
dr_Zfhx4      LQLHQQGLPPDEDNLADIFLVKDCPPNESEEPGEDS----------------------E  1103
              ********  :****::**:**:** ** *   . .:

hs_ZFHX4      GAIKPTAVAEDDEKDTSERDNSEGKNSNKDSGIITPEKELKVSVAGGTQPLLLAKEEDVA  1199
mm_ZFHX4      DPLKPPTVAEDDEKEAHKRDNSEGKISTKDP-VIVPEKELKV-VTGATQPLLLAKEDNTG  1195
dr_Zfhx4      EFPQASSLAAAEDKDSSKRDAIEGKTTEKDSRINTLAKETLGIATAGKQSLQQEKENDTP  1163
                 :  ::*  ::*:: :**  *** : **  : .  **    .:...* *   **::.

hs_ZFHX4      TKRSKPTEDNKFCHEQFYQCPYCNYNSRDQSRIQMHVLSQHSVQPVICCPLCQDVLSNKM  1259
mm_ZFHX4      TKRSKPTEDNKFCPEQFYQCPYCNYNSRDQSRIQMHVLSQHSVQPVICCPLCQDVLSNKM  1255
dr_Zfhx4      PKRPKSAEEKTLSNEQVQQCPYCNYSNKDANRLQLHIMSQHSMQPVISCPLCQDVLSNKI  1223
               ** * :*::.:. **. *******..:* .*:*:*::****:****.***********:

hs_ZFHX4      HLQLHLTHLHSVSPDCVEKLLMTVPVPDVMMPNSMLLPAAASEKSERDTPAAVTAEGSGK  1319
mm_ZFHX4      HLQLHLTHLHSVSPDCVEKLLMTVPVPDVMMPNSMLLPAAAPEKSEQDPPTALTAEGSGK  1315
dr_Zfhx4      HLQLHLTHLHSVAPDCVEKLLMTVAGPDLPVPSSLLSASLQDKVPSLMDTSAVNPEGSGK  1283
              ************:***********  **: :*.*:*  :    :   .    :*:. *****

hs_ZFHX4      YSGESPMDDKSMAGLEDSKANVEVKNEEQKPTKEPLEVSEWNKNSSKDVKIPDTLQDQLN  1379
mm_ZFHX4      YSGDSPVDDKSMSGLEDSKVGVEIKNEEQKPAKEPVEASEWNKTSSKDVNISDALQDQLN  1375
dr_Zfhx4      TMGNSSKDLKNGT--GQDKSEVDLTSEELKPLKEAAEAPDWKKASGQDRKSPDALQEHLS  1341
                *:*  * *. :   :.*  *::..** ** **  *. :*:* *.:* :  *:**::*.

hs_ZFHX4      EQQKRQPLSVSDRHVYKYRCNHCSLAFKTMQKLQIHSQYHAIRAATMCNLCQRSFRTFQA  1439
mm_ZFHX4      EQQKRQPLSVSDRHVYKYRCNHCSLAFKTMQKLQIHSQYHAIRAATMCTLCQRSFRTFQA  1435
dr_Zfhx4      DLQKRQQLSVSDRHVYKYRCNHCSLAFKTMQKLEIHSQYHAIRAATMCTLCQRSFRTFLA  1401
              : **** *************************:**************.********* *

hs_ZFHX4      LKKHLEAGHPELSEAELQQLYASLPVNGELWAESETMSQDDHGLEQEMEREYEVDHEGKA  1499
mm_ZFHX4      LKKHLEAGHPELSEAELQQLYASLPMNGELWAESETMTQDDHGIDQEMEREYEVDHEGKA  1495
dr_Zfhx4      LRKHLETGHPELTEAEVQQLCGNLPLNGDISESEMRALEEAQAFENELDKDEELDQEGKA  1461
              *:****:*****:***:*** ..**:**::  ..    :: :.:::*:::: *:*:****

hs_ZFHX4      SPVGSDSSSIPDDMGSEPKRTLPFRKGPNFTMEKFLDPSRPYKCTVCKESFTQKNILLVH  1559
mm_ZFHX4      SPVESDSSSIPDDLGLEPKRTLPFRKGPNFTMEKFLDPSRPYKCTVCKESFTQKNILLVH  1555
dr_Zfhx4      SPTGSDSSSLLDDMGSEPKRTLPFRKGPNFTMEKFLDPSRPYKCTVCKESFTQKNILLVH  1521
              **. *****: **:* ********************************************

hs_ZFHX4      YNSVSHLHKLKKVLQEASSPVPQETNSNTDNKPYKCSICNVAYSQSSTLEIHMRSVLHQT  1619
mm_ZFHX4      YNSVSHLHKLKKVLQEASSPVPQEANSSTDNKPYKCSTCSVAYSQSSTLEIHMRSVLHQT  1615
dr_Zfhx4      YNSVSHLHKLKKVIQEASSPVPQETSNNVDNKPYKCNICNVAYSQSSTLEIHMRSVLHQT  1581
              *************:**********:....*******. *.*******************

hs_ZFHX4      KARAAKLEPSGHVAGGHSIAAN---VNSPGQGMLDSMSLAAV-NSKDTHLDAKELNKKQT  1675
mm_ZFHX4      KARAAKLEPSRHLPSGHSITAA---VNSPGQGMLESMSLASV-NSKDTHLDAKELNKKQT  1671
dr_Zfhx4      KARTAKLETSTSTGSGSSGSTSSKSPVPPNHGNTDSASAAPVSTNKENTVDAKEVTMKQT  1641
              ***:**** *     .* * ::        *.:*  :* * * * ..*:. :****:. ***

hs_ZFHX4      PDLISAQPAHHP-PQSPAQIQMQLQHELQQQAAFFQPQFLNPAFLPHFPMTPEALLQFQQ  1734
mm_ZFHX4      PELISAQPTHHPPPRSPAQIQMQLQHELQQQAAFFQPQFLNPAFLPHFPMTPEALLQFQQ  1731
dr_Zfhx4      TEHIPVQPN-HPPTQSPAQLQMQLQHELQQQAAFFQPQFLNPAFFPHFPMTPEALLQFQQ  1700
               : * .**  **  :****:************************:***************

hs_ZFHX4      PQFLFPFYIPGTEFSLGPDLGLPGSATFGMPGMTGMAGSLLEDLKQQIQTQHHVGQTQLQ  1794
mm_ZFHX4      PQFLFPFYIPGAEFSLGPDLGLPTSTTFGVPGMTGMAGSLLEDLKQQIQTQHHVGQTQLQ  1791
dr_Zfhx4      PQFLFPFYIPGAEFNISPELALHS-AAFGMPG---MTGSFLEDLKQQMQQQHQLGQQQQL  1756
              ***********:**.:.*:*.*   ::**:**   *:**:*******:* **::** *

hs_ZFHX4      ILQQQAQQYQATQPQLQPQKQQQQPPPPQQQQQQQASKLLKQEQSNIVSADCQIMKDVPS  1854
mm_ZFHX4      FLQ-QAQQYQAVQPQLQPQNQQ-PPLPQQQQPQQQPSKLLKQEQGSLASTDCQLMKDMPS  1849
dr_Zfhx4      QLSQQQAQQQASQSQMQQQKVQQQSHKPKTESN------------HNVLSEIQMSRD---  1801
               *. *  * ** * *:* *: *       : : :            . :: *: :*

hs_ZFHX4      YKEAEDISEKPEKPKQEFISEGEGLKEGKDTKKQKSLEPSIPPPRIASGARGNAAKALLE  1914
mm_ZFHX4      YKEAEEVTEKQEKPKQEFINDTEGLKDSKDIKKQKSLEPCIPPPRIASGARGNAAKALLE  1909
dr_Zfhx4      --AEEHLEKQEGKAKQDTANENDNGKDAKDNRKPKFSEPLIPPPRIISGARGNAAKALLE  1859
                  *.: ::  * **:  .: :. *:.** :* *  ** ****** ************

hs_ZFHX4      NFGFELVIQYNENRQKVQKKGKSGEGENTDKLECGTCGKLFSNVLILKSHQEHVHGQFFP  1974
mm_ZFHX4      NFGFELVIQYNENRQKVQKKGKSGEGENSDKLECGICGKLFSNVLILKSHQEHVHGQFFP  1969
```

Index Family BN00337
p.(Lys1035_Asp3612delinsAsn)

Family BN00648
c.3964+2T>A
splice donor

```
dr_Zfhx4          NFGFELVIQYNENRQKSQKKNK--EDELTDKLECGLCGKLFSNMLILKSHQEHIHGQFFP  1917
                  **************** ***.*  *.* :****** *******:*********:******

hs_ZFHX4          YAALEKFARQYREAYDKLYPISPSSPETPPPPPPPPPLPPAPPQPSSMGPVKIPNTVSTP  2034
mm_ZFHX4          YGALEKFARQYREAYDKLYPISPSSPETPPPPPPPPPLPPAPPQPSTLGPVKIPNTVSAP  2029
dr_Zfhx4          YVELEKFAQQYREAYDKLYPINPASPETPPPPPPPPPPPPPAPLNTL-PANA--SVG-K  1973
                  *  *****:************.*:************* ** *   .:: *.:   :*.

hs_ZFHX4          LQAPPPTPPPPPPPPPPP-PPPPPPPPPSAPPQVQLPVSLDLPLFPSIMMQPVQHPALPP  2093
mm_ZFHX4          LQAPPPTPPPPPPPPPPPPPPPPPPPPPSAPQQVQLPVSLDLPLFPSIMMQPVQHPALPP  2089
dr_Zfhx4          SQTPSPAPI-QTPQQAPQPPPPPPPPPPTAPPQVQLPVSLDMPIFPPLMMQSVQHPGLPP  2032
                   *:* *:*    *   *  *********:** *********:*:** :*** ****.***

hs_ZFHX4          QLALQLPQMDALSADLTQLCQQQLGLDPNFLRHSQFKRPRTRITDDQLKILRAYFDINNS  2153
mm_ZFHX4          QLALQLPQMDTLSADLTQLCQQQLGIDPNFLRHSQFKRPRTRITDDQLKILRAYFDINNS  2149
dr_Zfhx4          QLALQLPTMDSLSSDLTQLCQQQLGLDPNFLRQSQFKRPRTRITDDQLKILRAHFDINNS  2092
                  ******* **:**:***********:******:********************:******

hs_ZFHX4          PSEEQIQEMAEKSGLSQKVIKHWFRNTLFKERQRNKDSPYNFSNPPITVLEDIRIDPQPT  2213
mm_ZFHX4          PSEEQIQEMAEKSGLSQKVIKHWFRNTLFKERQRNKDSPYNFSNPPITVLEDIRIDPQPT  2209
dr_Zfhx4          PNEEQIQEMADKSGLPQKVIKHWFRNTLFKERQRSKDSPYNFNIPPMTTLEDIRLESQVN  2152
                  *.********:**** ******************.*******. **:*.*****:: * .

hs_ZFHX4          SLEHYKSDASFSKRSSRTRFTDYQLRVLQDFFDTNAYPKDDEIEQLSTVLNLPTRVIVVW  2273
mm_ZFHX4          SLEHYKSDAAFSKRSSRTRFTDYQLRVLQDFFDTNAYPKDDEIEQLSTVLNLPTRVIVVW  2269
dr_Zfhx4          LLEYTRIDSASNKRSSRTRFTDYQLRVLQDFFDTNAYPKDDEIEQLSTVLNLPTRVIVVW  2212
                   **: : *:: ************************************************

hs_ZFHX4          FQNARQKARKSYENQAETKDNEKRELTNERYIRTSNMQYQCKKCNVVFPRIFDLITHQKK  2333
mm_ZFHX4          FQNARQKARKSYENQAEAKDNEKRELTNERYIRTSNMQYQCKKCNVVFPRIFDLITHQKK  2329
dr_Zfhx4          FQNARQKARKTYENQADTKESEKKELTNERYIRTNNMQYQCKKCSVIFPRIFDLITHQKK  2272
                  **********:*****::*:.**:**********.*********.*:*************

hs_ZFHX4          QCYKDEDDDAQDESQTEDSMDATDQVVYKHCTVSGQTDAAKNAAAPAASSGSGTSTPLIP  2393
mm_ZFHX4          QCYKDEDDDAQDESQTEDSMDATDQVLYKHCMVSGQTDTAKSTATLVASSGSGTSTPLIP  2389
dr_Zfhx4          QCYKDEDDEAGDEHILEDLSENIEQSLAKPME------AAKQ--SFVTASSSGSSSPLMP  2324
                  ********:* **   **  :  :* : *         :**.  : .::*.**:*:**:*

hs_ZFHX4          SPKPEPEKTSPKPEYPAEKPKQSDPSPPSQGTKPALPLASTSSDPPQASTAQPQPQPQPP  2453
mm_ZFHX4          SPKPEPEKNSPKTEYPGEKTKQSDPSLPQG-TKSAPSSVLTSSEPQQA--SIPQPPTQPP  2446
dr_Zfhx4          SPRPDIGKTSPKPELLNEKSKLGETAPLQTPKNPNEL------KPSKAS--TPQPLSQKV  2376
                  **:*:  *.*** *   ** * .: :  .  .:          .* :*    ***  *

hs_ZFHX4          KQPQLIGRPPSASQTPVPSSPLQISMTSLQNSLPPQLLQYQCDQCTVAFPTLELWQEHQH  2513
mm_ZFHX4          KQPQLIGRPPSASQTPIPSSPLQISMTSLQNSLPPQLLQYPCDQCTIAFPTLELWKEHQH  2506
dr_Zfhx4          PQPQLSRPHSQPQSTPVPSSPLSLALSSLNNSLPPQMLQYHCEQCKIAFPTVELWQEHQH  2436
                   ****     . ..**:*****.::::**:******:*** *:**.:****:***:****

hs_ZFHX4          MHFLAAQNQFLHSPFLERPMDMPYMIFDPNNPLMTGQLLGSSLTQMPPQASSSHTTAPTT  2573
mm_ZFHX4          MHFLAAQNQFLHSPFLERPMDMPYMIFDPNNPLMTGQLLGSSLTQMPPQTSTAHTTAPAS  2566
dr_Zfhx4          MHFLAAQNQFLHSQFLERPLDMPYMIFDPNNPLMTGQLLSGALSQIPTASNSGLT----M  2492
                  ************* *****:*******************..:*:*:*  :.:. *

hs_ZFHX4          VAASLKRKLDDKEDNNCSEKEGGNSGEDQHRDKRLRTTITPEQLEILYEKYLLDSNPTRK  2633
mm_ZFHX4          VAASLKRKLEDKEDNNCSEKEGGNSGEDQHRDKRLRTTITPEQLEILYEKYLLDSNPTRK  2626
dr_Zfhx4          NSNSLKRKLDEKEDG-SHEKDGLNSAEDQHRDKRLRTTITPEQLEILYDKYLLDSNPTRK  2551
                   : ******::***. . **:* **.**********************:***********

hs_ZFHX4          MLDHIAREVGLKKRVVQVWFQNTRARERKGQFRAVGPAQSHKRCPFCRALFKAKSALESH  2693
mm_ZFHX4          MLDHIAREVGLKKRVVQVWFQNTRARERKGQFRAVGPAQSHKRCPFCRALFKAKSALESH  2686
dr_Zfhx4          MLDHIAHEVGLKKRVVQVWFQNTRARERKGQFRAVGPSQTHKKCPFCRALFKAKSALDSH  2611
                  ******:******************************:*:**:**************:**

hs_ZFHX4          IRSRHWNEGKQAGYSLPPSPLISTEDGGESPQKYIYFDYPSLPLTKIDLSSENELASTVS  2753
mm_ZFHX4          IRSRHWNEGKQAGYSLPPSPLISTEDGGESPQKYIYFDYPSLPLTKIDLSTENELASTVS  2746
dr_Zfhx4          IRSRHWHEAKQAGFSLPPSPMMPQDDEGQSPHKYSFSDYLQMPTKI-EMN-ENEPPTASS  2669
                  ******:*.****:******::  :* *:**:** : ** .:* .  ::. ***  :: *

hs_ZFHX4          TPVSKTAELSPKNLLSPSSFKAECSEDVENLNAPPAEAGYDQNKTDFDETSSINTAISDA  2813
mm_ZFHX4          TPVSKTAELSPKNLLSPSSFKAECPEDVENLNAPSADAGYDQSKTDFDETSSINTAISDA  2806
dr_Zfhx4          TPVK-PSETLFKNFLNIPSLKAEHSDELEALNMSSAEASFDTNKMDFDETSSINTAVSDA  2728
                  ***.  :*   **:*.  *:***  :::* **   *:*.:* .* ***********:***

hs_ZFHX4          TTGDEGNTEMESTTGSSGDVKPALSPKEPKTLDTLPKPATTPTTEVCDDKFLFSLTSPSI  2873
mm_ZFHX4          TTGDEGAADMENT-GGSGEVKPALSPKETKTLDSLQKPATTPTTEVCDDKFLFSLTSPSI  2865
dr_Zfhx4          TTGDETNNEVENLTINGSEKM----------I--ENKSNQAQTSDMNEDRFPFNMVSPAL  2776
                  *****   ::*.   ...:            :    *   : *::: :*:* *.:.**::

hs_ZFHX4          HFNDKDGDHDQSFYITDDPDDNADRSETSSIADPSSPNPFGSSNPFKSK--SNDRPGHKR  2931
mm_ZFHX4          HFNDKDGDHDQSFYITDDPDDNADRSETSSIADPSSPNPFGSSNPFKSK--SNDRPGHKR  2923
dr_Zfhx4          SFSGKDSD--YFNYRDDDFDDNADRSETSSLADPSSPSPFGSTNPFKSSKAGGERPGHKR  2834
                   *..**.*     *  ** ***********:******.****:*****.  ..:******

hs_ZFHX4          FRTQMSNLQLKVLKACFSDYRTPTMQECEMLGNEIGLPKRVVQVWFQNARAKEKKFKINI  2991
mm_ZFHX4          FRTQMSNLQLKVLKACFSDYRTPTMQECEMLGNEIGLPKRVVQVWFQNARAKEKKFKINI  2983
dr_Zfhx4          FRTQMSNLQLKVLKACFSDYRTPTMQECEMLGNEIGLPKRVVQVWFQNARAKEKKFKINI  2894
                  ************************************************************

hs_ZFHX4          GKPFMINQGGTEGTKPECTLCGVKYSARLSIRDHIFSKQHISKVRETVGSQLDREKDYLA  3051
mm_ZFHX4          GKPFMINQSGTDGTKPECTLCGVKYSARLSIRDHIFSKQHISKVRETVGSQLDREKDYLA  3043
```

```
dr_Zfhx4          GKPFMISQGSPDGPRPECTLCGVKYTARLSIRDHIFSKQHIAKVQETLGNQVDREKDYLA  2954
                  ******.*.. :* :**********:***************:**:**:*.*:********

hs_ZFHX4          PTTVRQLMAQQELDRIKKASDVLGLTVQQPGMMDSSSLHGISLPTAYPGLPGLPPVLLPG  3111
mm_ZFHX4          PTTVRQLMAQQELDRIKKASDVLGLTVQQQGITDNCSLHGISLQAAYPGLPGLPPVILPG  3103
dr_Zfhx4          PTTVRQLMAQQELDRLKKATDVLNLPAQQQPAVDNNALHGLSLPTAYPGISGLPPVLLPG  3014
                  ***************:***:***.* .**    *. :***:** :****: *****:***

hs_ZFHX4          MNGPSSLPGFPQNSNTLTPPGAGMLGFPTSATSSPALSLSSAPTKPLLQTPPPPPPPPPP  3171
mm_ZFHX4          MNGPSSLPGFPQNSNTLTSPGTGMLGFPSSATSSPALSLSSGPTKSLLQTPPPPPPPP--  3161
dr_Zfhx4          VNGPSSLPGFPPNTPALASPGAGMLGFPTPATPSPAMSLSSTPTKTLLQTPPPPPPPQPS  3074
                  :********** *: :*: **:******: ** ***:**** *** ***********

hs_ZFHX4          ---PPSSSLSGQQTEQQNKESEKKQTKPNKVKKIKEEELEATKPEK-HPKKEEKISSALS  3227
mm_ZFHX4          ---PPPSSLSGQQTEPQNKESEKKQTKPNKVKKIKEEESEAIKPEK-HPKKEEKISSALT  3217
dr_Zfhx4          ALPLPPTPLAANQTEQHIKESEKDKKL--EKPKVKEREADTARPETPIVKKKEKPSPPLS  3132
                      * : *:.:*** : *****.:.   :  *:**.* :: :**.   **:** *  *:

hs_ZFHX4          VLGKVVGETHVDPIQLQALQNAIAGDPASFIGGQFLPYFIPGFASYFTPQLPGTVQGGYF  3287
mm_ZFHX4          VLGKVVGETHMDPTQLQALQNAIAGDPASFIGGQFLPYFIPGFASYFSPQLPGTVQGGYL  3277
dr_Zfhx4          MLGKLGSEGQMDPAQLQALQNAIAGDPGSFLGGQFLPYFIPGFASCFSPQLPGGVQGGYF  3192
                  :***: .* ::** *************.**:************** *:***** *****:

hs_ZFHX4          PPVCGMESLFPYGPTMPQTLAGLSPGALLQQYQQYQQNLQESLQKQQKQQQEQQQKPVQA  3347
mm_ZFHX4          PPICGMESLFPYGPAVPQTLAGLSPGALLQQYQQYQQSLQDSLQKQQKQQQEQQQKPVPA  3337
dr_Zfhx4          PPLCGMENLFPYGPAMPQAIAGLSPTALLQQYQQYQQSLQDSLQKQQHQQQQQQKQSEPQ  3252
                  **:****.******::**::***** ***********.**:******:***:**::

hs_ZFHX4          KT--SKV----ESDQPQNSNDASETKEDKSTATESTKEEPQLESKSADFSDTYVVPFVKY  3401
mm_ZFHX4          KT--AKG----EGDQPQSSNEASETKEEKSTAPESTKEEVQLDSKSAEFSDTCIVPFVKY  3391
dr_Zfhx4          RKTPAPMKSTSAQSNSLKPKETVETKDDKGSSTESTNEEPQTNTKSSGFPDAFIVPSIKH  3312
                  :.  :        .:  . ::: ***::*.:: ***:** * ::**: * *: :** :*:

hs_ZFHX4          EFICRKCQMMFTDEDAAVNHQKSFCYFGQPLIDPQETVLRVPVSKYQCLACDVAISGNEA  3461
mm_ZFHX4          EFVCRKCQMMFTDEDATVNHQKSFCYFGQPLIDPQETVLRIPVSKYQCLACDLALSGNEA  3451
dr_Zfhx4          EFICRKCQMIFADEDSAARHQKSFCYFGHPFIDPQETVLRKAVSKYNCIACNVSVSGNEA  3372
                  **:******:*:***::..*********:*:*********  ****:*:**::::*****

hs_ZFHX4          LSQHLQSSLHKEKTIKQAMRNAKEHVRLLPHSVCSPNPNTTSTSQSAASSNNTYPHLSCF  3521
mm_ZFHX4          LSQHLQSSLHKEKTIKQAMRNAKEHVRLLPHSVCSPPPNTSSTSPSAASSNNTYPHLSCF  3511
dr_Zfhx4          LGQHLQSSLHKEKTIKQAMRNAKEHARLLPHSVCSPNPNTTSTSQSAASSNNTLPHLSHL  3432
                  *.***********************.********** ***:*** ******** **** :

hs_ZFHX4          SMKSWPNILFQASARRAAS-PPSSPPSLSLPSTVTSSLCSTSGVQTSLPTESCSDESDSE  3580
mm_ZFHX4          SMKSWPNILFQASARKAAS-SPSSPPSLSLPSTVTSSLCSTSGVQTSLPTESCSDESDSE  3570
dr_Zfhx4          SMKSWPNILFQASARKAASCPSASPPPLSLPSTVTSTSCSTSGVQTSLPTESCSDESDSE  3492
                  ***************:***   :*** *********: **********************

hs_ZFHX4          LSQKLEDLDNSLEVKAKPASGLDGNFNSIRMDMFSV  3616
mm_ZFHX4          LSQKLQDLDNSLEVKAKPASGLDGNFNSVRMDMFSV  3606
dr_Zfhx4          LSQKLDDLDNALEAKAKAASGLDGNFSSMRMDMFSV  3528
                  *****:****:**.*** ********.*:*******
```

Figure S1. Conservation of *ZFHX4* on amino acid level created with Clustal Omega (Madeira et al., 2022). (A) ZFHX4 amino acid sequence identity between human (hs) mouse (mm) and zebrafish (dr) in percent. UniProt sequence identifier given in brackets. **(B)** ZFHX4 protein sequence alignment (hs, mm and dr). Amino acid residues are colored corresponding to their physiochemical features: red - small, hydrophobic (including aromatic, without Y); blue - acidic; purple - basic (without H); green - hydroxyl, sulfhydryl, amine and G. Asterisk, full conservation in that position; colon or period, conservation between residue groups with strongly (:) or weakly (.) similar features. Positions of zinc finger (grey) and homeobox (yellow) domains are highlighted in hs ZFHX4 sequence. Variant locations from present study, Zametica et al. and Girisha K.M. & colleagues are marked.

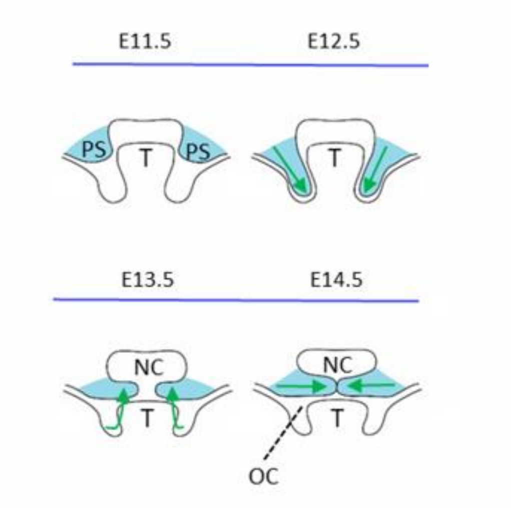

Figure S2. Timeline of secondary palate development in mice (E11.5–E14.5). Anatomical structures that were dissected for the bulk RNA-Seq dataset (Figure 2A) are given in light blue. PS, palatal shelves; T, tongue; NC, nasal cavity; OC, oral cavity.

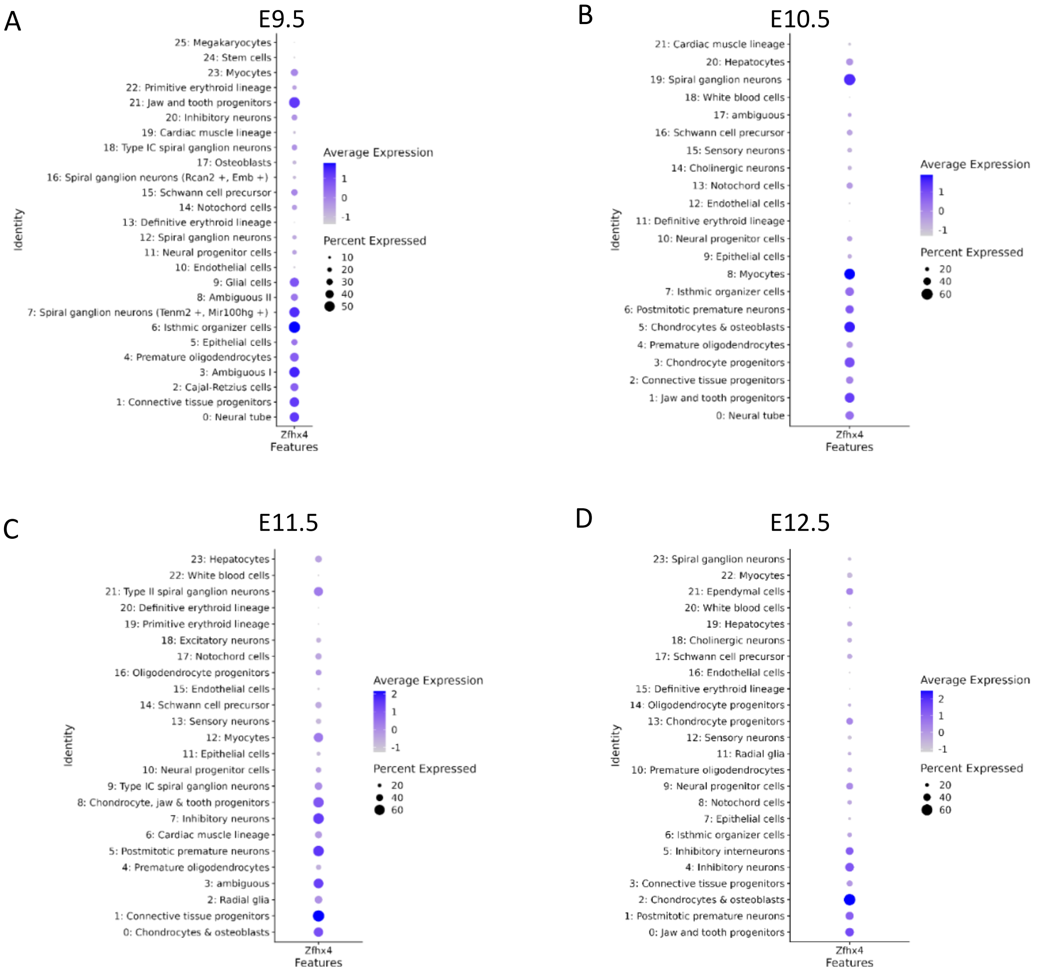

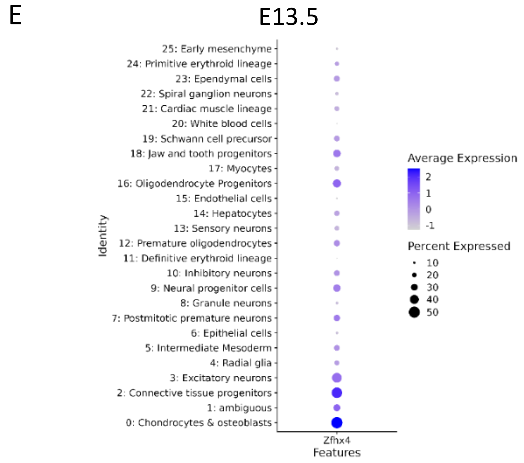

Figure S3. Expression pattern of *Zfhx4* in mouse whole embryo scRNA-Seq data from different embryonic stages (Siewert et al. 2023). The color of the dots corresponds to the average scaled expression level. The size of the dots corresponds to the percentage of cells that express the gene in the respective cell type. **(A-E)** Embryonic days E9.5 to E13.5 in 1-day increments.

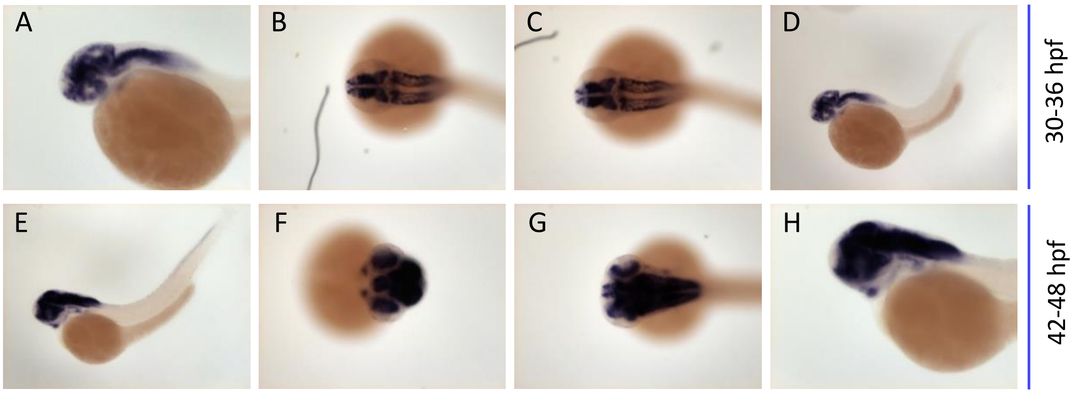

Figure S4. Whole mount *in situ* hybridization of *zfhx4* in zebrafish larvae (zfl) of different developmental stages from Thisse, B., Thisse, C. (2004) Fast Release Clones: A High Throughput Expression Analysis. ZFIN Direct Data Submission. (http://zfin.org). (A-D) Zfl from Prim 15 – Prim 25 stage (30-36 hpf). Expression according to Thisse, B., Thisse, C., 2004 annotations in diencephalon, hindbrain, midbrain and telencephalon as indicated by blue/purple dye. (**E-H)** Zfl from High-Pec – Long Pec stage (42-48 hpf). Expression according to Thisse, B., Thisse, C., 2004 annotations in brain, pharyngeal arch and retinal ganglion cell layer as indicated by blue/purple dye. Zfl, Zebrafish larvae; hpf, hours post-fertilization.

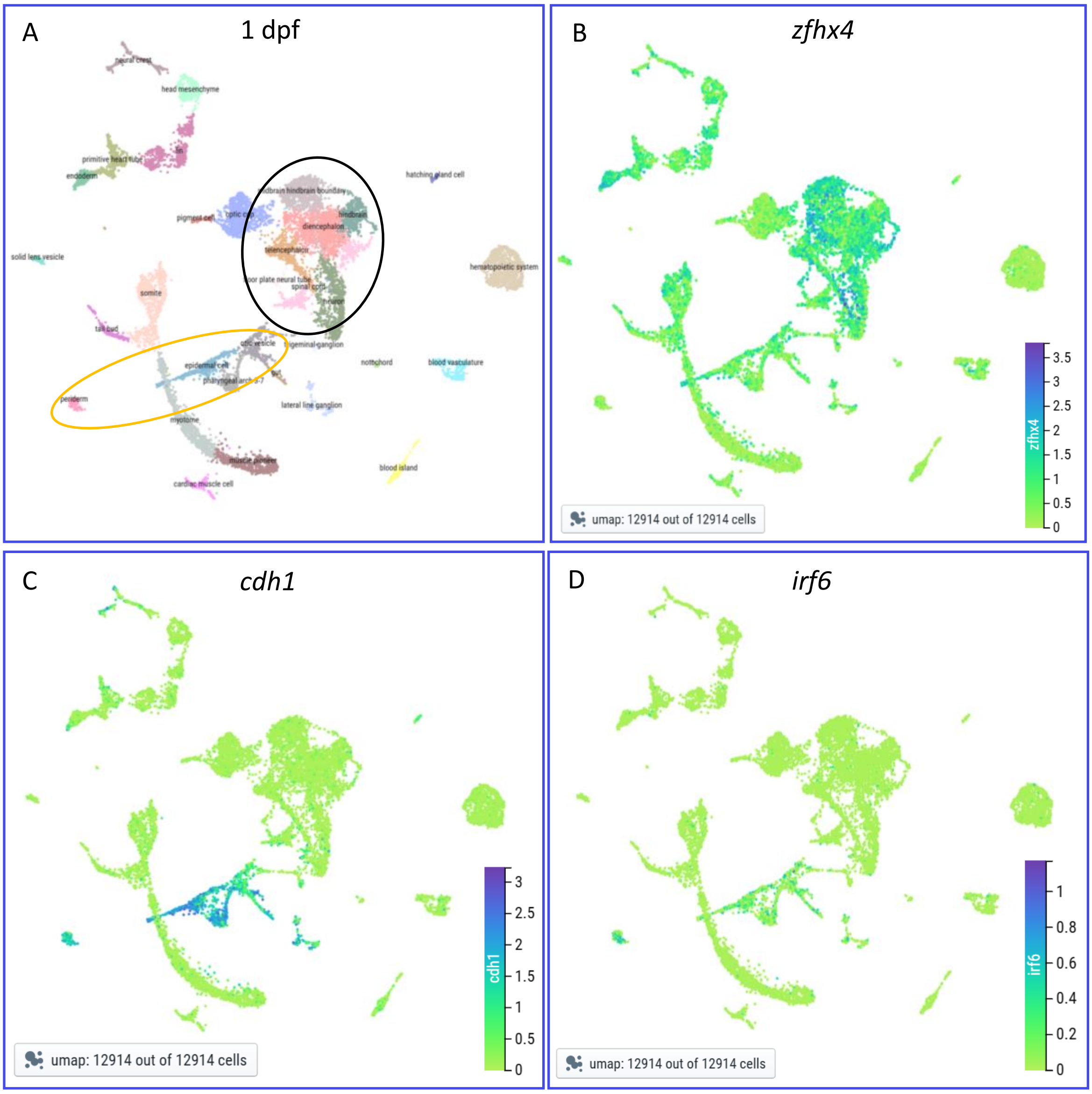

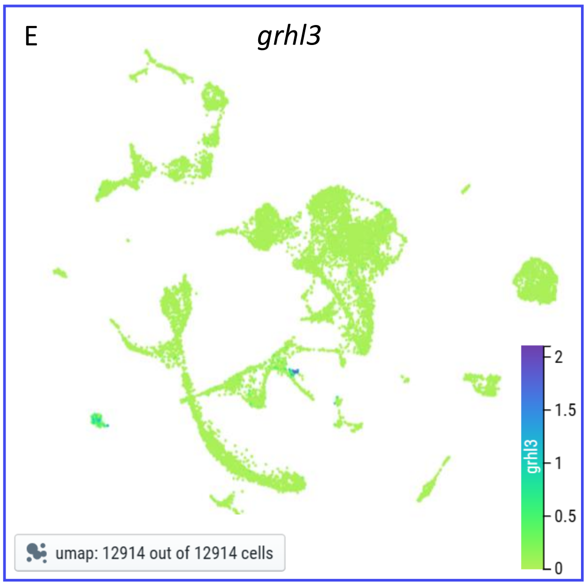

Figure S5. UMAP representation of single cell RNA-Seq data from 1 dpf single-embryo transcriptomes of zebrafish (from Zebrahub, Lange et al. 2023, bioRxiv). (A) UMAP of 1 dpf old zebrafish embryo colored by annotated cell types. **(B)** UMAP colored by expression of *zfhx4* ranging from no (light green) to strong expression (purple). Cell clusters are the same as depicted in A. **(C-E)** Same as in B for the established clefting genes **(C)** *cdh1,* **(D)** *irf6 and* **(E)** *grhl3*. The expression of *cdh1*, *irf6* and *grhl3* at 1 dpf is mainly in epidermal and peridermal cells (orange circle in A). The expression pattern of *zfhx4* is much broader than the one for the other established clefting genes as it is also expressed in different neuronal cell types (black circle in A). dpf, days post fertilization.

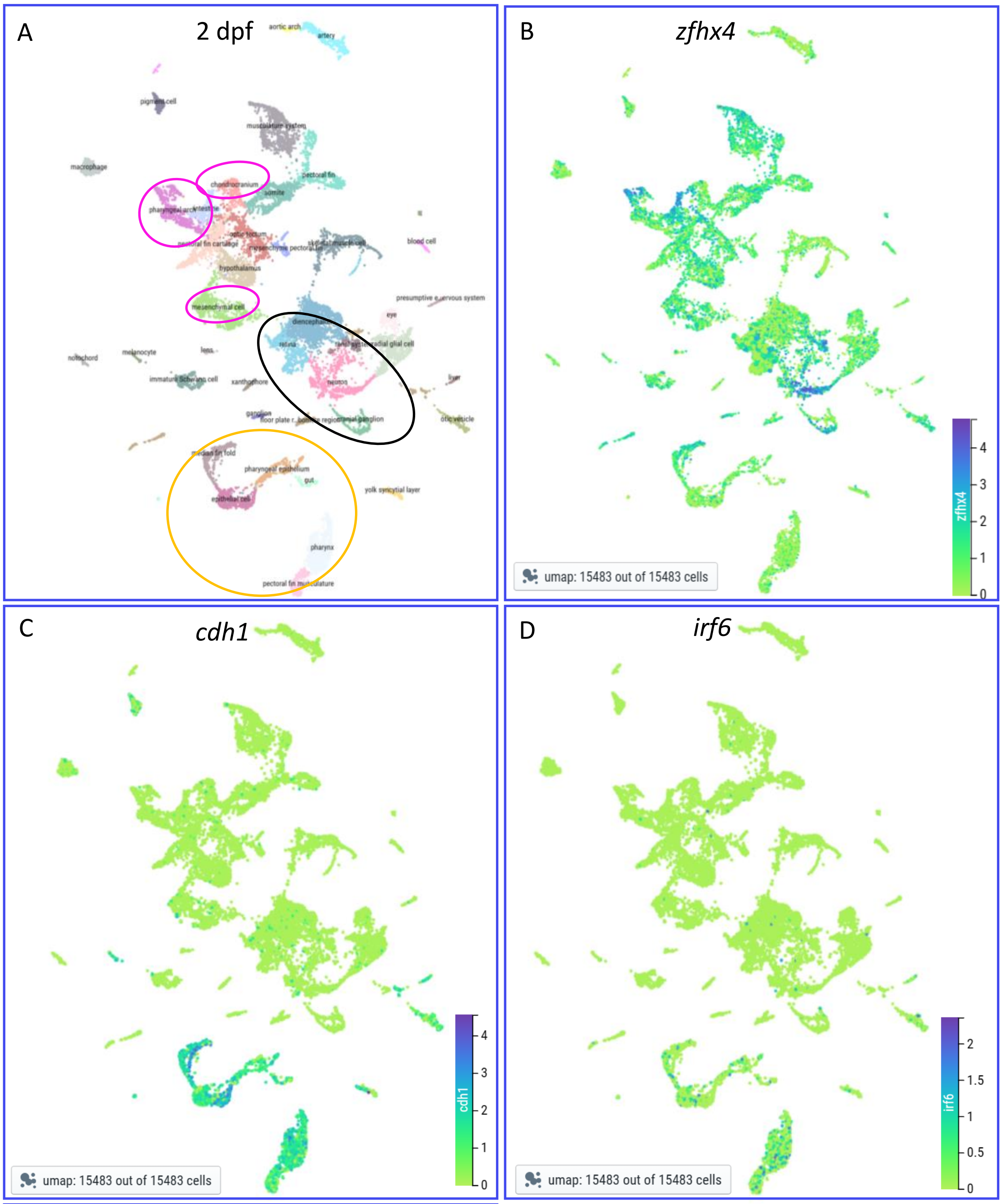

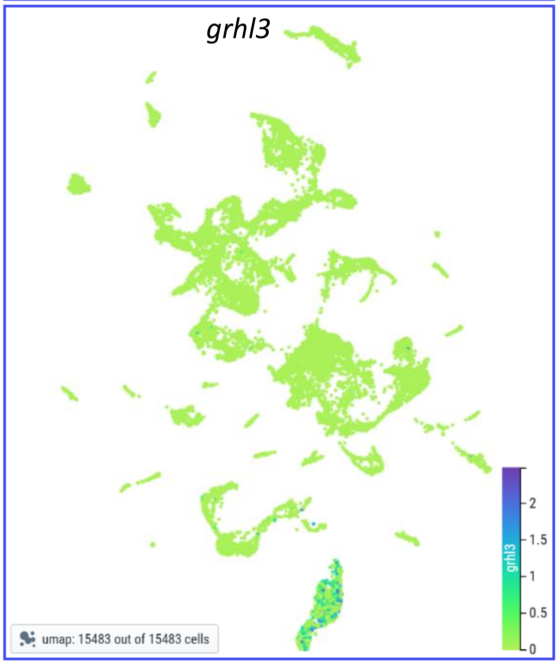

Figure S6. UMAP representation of single cell RNA-Seq data from 2 dpf single-embryo transcriptomes of zebrafish (from Zebrahub, Lange et al. 2023, bioRxiv). (A) UMAP of 2 dpf old zebrafish embryo colored by annotated cell types. **(B)** UMAP colored by expression of *zfhx4* ranging from no (light green) to strong expression (purple). Cell clusters are the same as depicted in A. **(C-E)** Same as in B for the established clefting genes **(C)** *cdh1*, **(D)** *irf6 and* **(E)** *grhl3*. The expression of *cdh1*, *irf6* and *grhl3* at 2 dpf is mainly in epithelial and pharyngeal cells (orange circle in A). The *zfhx4* gene is broadly expressed, among others, in cell types important to craniofacial development like chondrocranial cells, mesenchymal cells and cells of the pharyngeal arches (pink circle in A) and neuronal cells (black circle in A). dpf, days post fertilization.

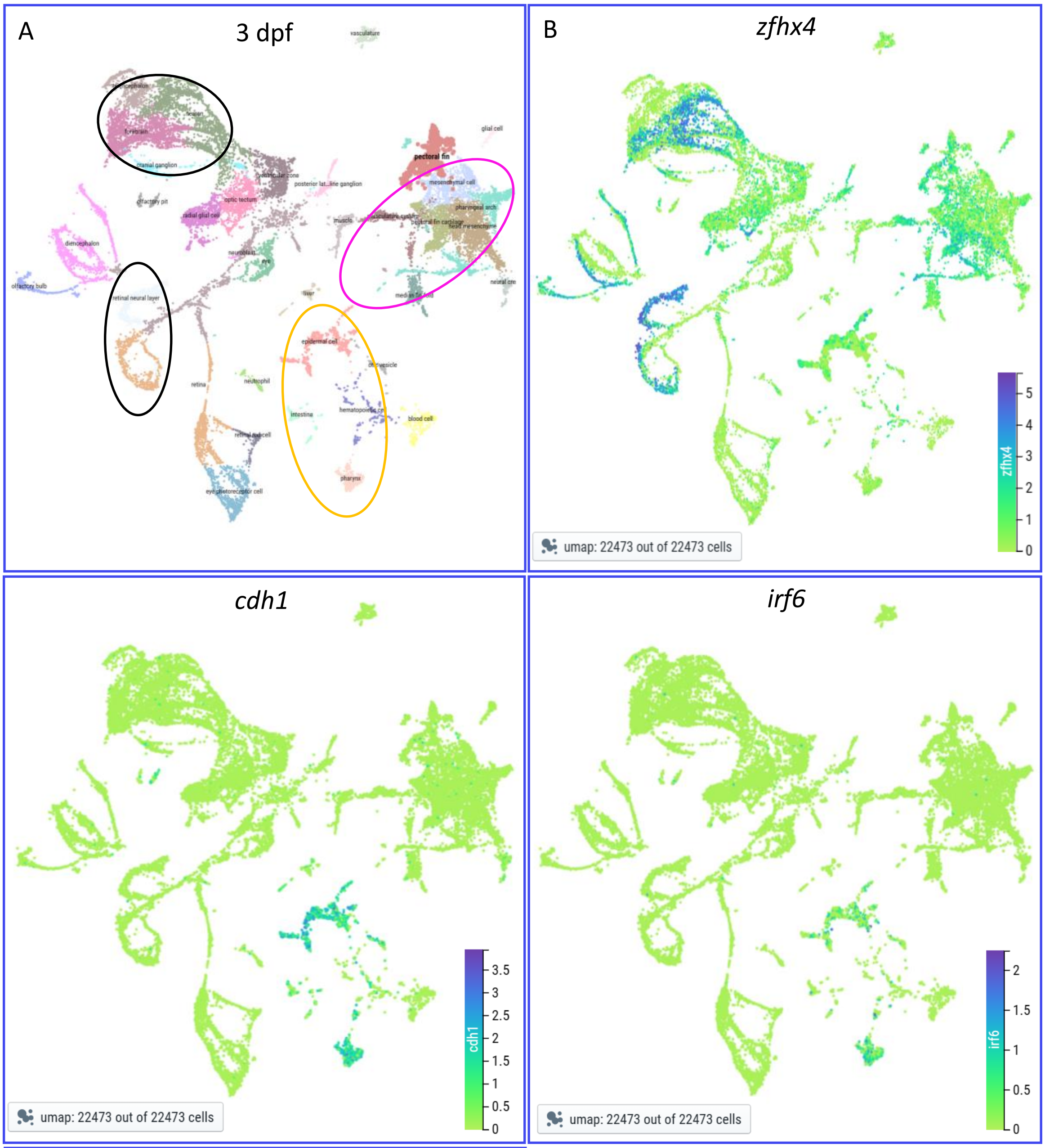

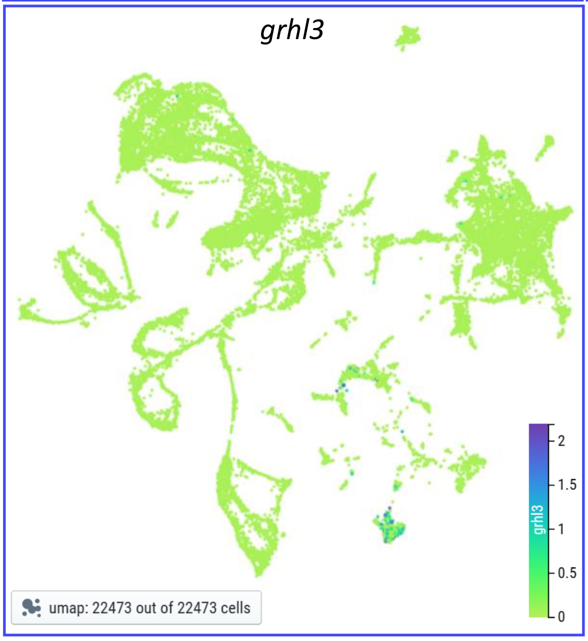

Figure S7. UMAP representation of single cell RNA-Seq data from 3 dpf single-embryo transcriptomes of zebrafish (from Zebrahub, Lange et al. 2023, bioRxiv). (A) UMAP of 3 dpf old zebrafish embryo colored by annotated cell types. **(B)** UMAP colored by expression of *zfhx4* ranging from no (light green) to strong expression (purple). Cell clusters are the same as depicted in A. **(C-E)** Same as in B for the established clefting genes **(C)** *cdh1,* **(D)** *irf6 and* **(E)** *grhl3*. The expression of *cdh1*, *irf6* and *grhl3* at 3 dpf is mainly in epithelial and pharyngeal cells (orange circle in A). The *zfhx4* gene is broadly expressed, among others, in cell types important to craniofacial development like head mesenchymal cells, mesenchymal cells and cells of the pharyngeal arches (pink circle in A) and also neuronal tissues (black circle in A). dpf, days post fertilization.

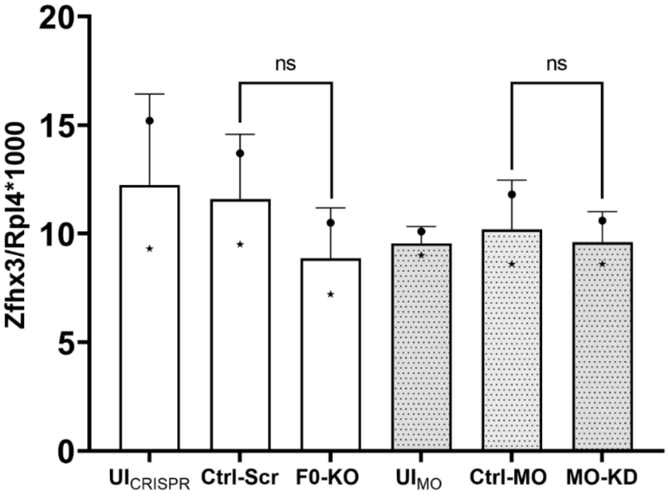

Figure S8. Detection of Zfhx3 with Liquid Chromatography Mass Spectrometry (LC-MS) analysis of protein lysates from heads of 3 dpf zfl (N=2) to show specificity of F0-KO and MO-KD. Depicted are Zfhx3 fold changes relative to 60S ribosomal protein L4 (Rpl4) abundance of each sample. Zfhx3 levels were not significantly decreased in F0-KO compared to Ctrl-Scr and in MO-KD compared to Ctrl-MO. Individual data of LC-MS run1 (stars) and run2 (dots) are plotted. Statistical testing was performed using two-way analysis of variance (ANOVA), Tukey's multiple comparisons test. Data are presented as means with standard deviation (SD). ns = not statistically significant. dpf, days post-fertilization; $UI_{CRISPR/MO}$, uninjected wildtype zfl; Ctrl-MO, Control MO injected zfl; Ctrl-Scr, Scrambled control injected zfl.
