# Supplementary material for: Role of ZFHX4 in orofacial clefting based on human genetic data and zebrafish models
Source: Eur J Hum Genet. 2024 Dec 19;33(5):595–606. doi: 10.1038/s41431-024-01775-9 (PMC7617551; doi:10.1038/s41431-024-01775-9)
Supplement: Supplementary file 3 — Table S1 [file 41431_2024_1775_MOESM3_ESM.pdf]

Table S1. Primer, morpholino, sgRNA and peptide sequences.

Primer CNV qPCR validation

	REGION (hg19)	SIZE	sequence F	sequence R
Primerpair1 (*ZFHX4*)	chr8:77690769-77690901	133 bp	ctctccaactcgagcctgtc	aaagatgtcaccgcatgcag
Primerpair2 (*ZFHX4*)	chr8:77733115-77733258	144 bp	caaacaggcaatgggtgtgt	gggaggagagttggcagagt
Primerpair3 (*ZFHX4*)	chr8:77776337-77776468	132 bp	agccaacacctccagtcaag	ctgcgaggtagatgtggtgt
BCN1	chr15:83931828-83931952	125 bp	TCAGTGCTTTGTCCAACAGG	GCAGATGTCACACTGGAAGC
CFTR	chr7:117232238-117232375	138 bp	GGAGATGCTCCTGTCTCCTG	GGGAGTCTTTTGCACAATGG

Morpholino oligonucleotides

Morpholino knock-down (MO-KD)	5'-GATCTCATTTCATCCAGCCTGTCAG-'3
Standard control morpholino (Ctrl-MO)	5'-CCTCTTACCTCAGTTACAATTTATA-'3

Primer CRISPR PCR

	REGION (danRer11)	SIZE	sequence F (Intron3)	sequence R (Intron4)
zfhx4	chr24:23300587-23301306	720 bp	CCTTTTGTTAGGGTGCCCTAC	CATACAGAACTAGGACAGTGGG

sgRNA sequences CRISPR (F0-KO)

name	start	end	strand	sgrna_seq
zfhx4_ex3_1	23300742	23300765	+	CAGGTGCACAGGATTGCCGA
zfhx4_ex3_2	23300781	23300804	-	AAGGTGGCAAAGCCAACCAG
zfhx4_ex3_3	23300920	23300943	-	GAGGAGTGGAGGGCCGTGAT
zfhx4_ex3_4	23300960	23300983	+	CCGTGGCCACGTGCCCGCTG
zfhx4_ex3_5	23301109	23301132	-	GCGGAGCTGCGTCTTGCGAG

Specific peptides for targeted Liquid Chromatography Mass Spectrometry

Zfhx4_1	GSASLASSADQSPR
Zfhx4_2	FSSDSLEALSGHVATER
Zfhx4_3	HQQSEGLR
Zfhx4_4	ETLGIATAGK
Zfhx4_5	VQETLGNQVDR
Zfhx3_1	CEVCDYETNVAR
Zfhx3_2	IHMTSEK
Zfhx3_3	FTDYQLR
Rpl4_1	SGQGAFGNMCR
Rpl4_2	NIPGITLQSVSR
Rpl4_3	LAPGGHIGR
Rpl4_4	LDDLYGTWR
Rpl4_5	VDYNLPMHK
Rpl4_6	MSITDLNR
